# Chitosan Oligosaccharides Suppress Adipogenesis and Lipid Accumulation in 3T3-L1 Preadipocytes via Multi-Pathway Transcriptomic Reprogramming

**DOI:** 10.3390/ijms27114970

**Published:** 2026-05-30

**Authors:** Sineenart Songkoomkrong, Siriporn Nonkhwao, Jirawat Saetan, Supawadee Duangprom, Prateep Amonruttanapun, Piyapon Janpan, Prasert Sobhon, Napamanee Kornthong

**Affiliations:** 1 Chulabhorn International College of Medicine, Thammasat University, Rangsit Campus, Pathumthani 12120, Thailand; sineenartsong@gmail.com (S.S.); siriphorn.nonkhaow@gmail.com (S.N.); su.duangprom@gmail.com (S.D.); wanderer_sci@yahoo.com (P.A.); piyapon.ater@gmail.com (P.J.); 2 Research Unit in Innovative Marine Biotechnology and Natural Bio-Resources for Sustainable Health and Wellness, Thammasat University, Pathumthani 12120, Thailand; 3 Division of Health and Applied Sciences, Faculty of Science, Prince of Songkla University, Hat Yai, Songkhla 90110, Thailand; jisaetan@gmail.com; 4 Department of Anatomy, Faculty of Science, Mahidol University, Bangkok 10400, Thailand; prasert.sob@mahidol.ac.th

**Keywords:** chitosan oligosaccharides (COS), 3T3-L1 preadipocytes, adipogenesis, lipid accumulation, transcriptomics

## Abstract

Obesity is a major global health burden that is linked to type 2 diabetes, cardiovascular disease, and metabolic syndrome. Chitosan oligosaccharides (COS) are bioactive compounds that are derived from the depolymerization of the chitosan in crustacean shells and are promising candidates for natural anti-adipogenesis effects. However, there is incomplete understanding of the molecular mechanisms by which structurally defined low-molecular-weight COS modulates adipogenic transcription networks and global transcriptional reprogramming. MALDI-TOF (matrix-assisted laser desorption/ionization time-of-flight) mass spectrometry and ^13^C NMR spectroscopy indicated a predominance of dimeric species (DP2) at *m*/*z* 344.79, which represents a lower molecular weight fraction and is proposed to improve the membrane permeability and intracellular bioavailability of COS. In a 3T3-L1 preadipocyte model, COS treatment at concentrations of 320–1280 µg/mL dose-dependently reduced intracellular lipid accumulation, triglyceride content, and adipocyte maturation while enhancing lipolysis and insulin-mediated glucose uptake. Western blot analysis indicated dose-dependent downregulation of PPARγ and C/EBPα. Transcriptomic RNA-seq analysis indicated large-scale transcriptional reprogramming with the altered expression of genes involved in PPAR signaling, PI3K-Akt, AMPK, insulin signaling, and fatty acid metabolism pathways among differentially expressed genes. These findings demonstrate that COS suppresses adipogenesis through the coordinated modulation of adipogenic transcription factors and multiple metabolic signaling pathways. The results support its potential as a promising natural compound but warrant preclinical investigation in the context of obesity and metabolic disorders.

## 1. Introduction

Obesity is characterized by elevated levels of adipose tissue associated with fat accumulation and adipocyte hypertrophy. It is a significant global health issue, and its prevalence is increasing, along with related metabolic diseases such as type 2 diabetes, cardiovascular disease, and metabolic syndrome. Thus, safe and efficacious natural anti-obesity substances have been sought after [1,2,3,4].

Chitosan oligosaccharides (COS) are low-molecular-weight derivatives that are obtained by the partial deacetylation and depolymerization of chitin, which is a naturally occurring (β1 → 4)-linked polysaccharide that is abundant in crustacean exoskeletons and fungal cell walls [5,6,7,8]. Structurally, COS contains varying proportions of *N*-acetyl-2-amine-2-deoxy-D-glucose (*N*-acetyl-glucosamine, GlcNAc) and 2-amino-2-deoxy-D-glucose (glucosamine, GlcN) residues, forming homo or heterooligomeric chains with a typical molecular weight below 2 kDa and a degree of polymerization (DP) under 20 [6,9]. When acetyl groups are removed, primary amino properties are revealed. At acidic to neutral pH, these groups are present in a protonated (-NH_3_+) form, which has high hydrophilicity, intestinal permeability, and a higher solubility than natural chitosan [10,11]. COS at molecular weights ranging from 300 to 700 Da (di- and tri-saccharides) exhibit optimal solubility and diffusion capabilities across mucosal barriers, which facilitate effective absorption and systemic distribution [12].

At the cellular level, COS with a molecular weight in the range of 1–10 kDa has strong anti-adipogenic effects. These effects result from the inhibition of the transcriptional activation of the peroxisome proliferator-activated receptor gamma (PPARγ) and the CCAAT/enhancer-binding protein alpha (C/EBPα), which are two key regulators of adipocyte differentiation [5,13]. Through downregulation of these adipogenic transcription factors, COS inhibits the transformation of preadipocytes to mature lipid-storing adipocytes and reduces lipid-droplet accumulation and intracellular triglyceride levels. In white and brown adipose tissues, COS with a molecular weight less than 1–3 kDa can inhibit the differentiation of 3T3-L1 preadipocytes into mature adipocytes and activate thermogenic genes such as *UCP1*, *PGC-1α*, and *PRDM16*, which results in greater energy expenditure and fatty acid oxidation [14,15]. The treatment of 3T3-L1 cells with COS dramatically reduces cell differentiation and the accumulation of lipids in a dose-dependent manner [5].

Proteomic analysis shows that COS treatment dysregulates several adipogenic molecules, including fatty acid-binding protein 4 (FABP4) and glucose transporter 4 (GLUT4). The inhibitory effect appears to be mediated through the C/EBPα and PPARγ pathways [16]. COS also decreases the expression of adipokines related to energy homeostasis and insulin responsiveness, including leptin, adiponectin, and resistin [5,7]. COS may also facilitate the browning of white adipose tissue and thermogenesis in brown adipose tissue by upregulating uncoupling protein 1 (UCP1) and peroxisome proliferator-activated receptor gamma coactivator-1 alpha (PGC-1α) through the activation of the p38 signaling pathway [15,17]. COS treatment significantly attenuates body weight gain and reduces fat accumulation in animals with obesity induced by a high-fat diet [7,15,17]. 

The phosphatidylinositol 3-kinase (PI3K)-protein kinase B (Akt) pathway and PI3K-PIP3 are critical for mediating adipogenesis. Akt activation by insulin stimulates various metabolic events, such as GLUT4 translocation to the plasma membrane for glucose uptake, lipogenic enzyme activation, and lipolysis inhibition [8]. Thus, the PI3K-Akt complex affects the expression of adipogenic transcription factors and may play an important role in connecting insulin signaling to the differentiation of adipocytes [18]. The dysregulation of these pathways leads to progressive obesity and obesity-related metabolic diseases [19].

The COS treatment of mice fed a high-fat diet leads to improvements in glucolipid metabolism disorder through the suppression of inflammation and the upregulation of PPARγ expression in HepG2 cells and liver tissues [20]. COS have also been reported to inhibit PPARγ and C/EBPα in adipocytes and to influence AMPK activation and PI3K-Akt signaling [21]. In hepatic steatosis models, COS treatment notably activates AMPK and reduces the expression of the lipogenic markers fatty acid synthase and sterol regulatory element-binding protein 1c (SREBP-1c). Simultaneously, it stimulates the expression of the fatty acid oxidation-related markers carnitine palmitoyltransferase 1A, acyl-coenzyme A oxidase 1, and PPARα. AMPK antagonist blocks the inhibition of lipogenesis and the increase in fatty acid oxidation induced by COS [22].

Nevertheless, the anti-adipogenic mechanisms of COS are still not fully understood, particularly in terms of their effects on global gene expression and signaling pathways in adipocytes [5]. In addition, the development of extraction methods and the attainment of COS with a well-defined low molecular weight are important challenges. Therefore, this study aimed to establish an extraction method for low-molecular-weight COS (<500 Da), as well as structural characterization using nuclear magnetic resonance (NMR) spectroscopy and MALDI-TOF (matrix-assisted laser desorption/ionization time-of-flight) mass spectrometry. The effects on adipocyte differentiation and lipid accumulation in 3T3-L1 cells were also validated. We hypothesized that COS mediates the suppression of adipocyte differentiation and triglyceride accumulation through the alteration of the PPARγ, PI3K-Akt, AMPK, and insulin signaling pathways, which were examined using a transcriptomic approach. This work illustrates the potential of low-molecular-weight COS as a promising natural compound that warrants preclinical investigation for its cellular and molecular mechanisms of action.

## 2. Results

### 2.1. MALDI-TOF Mass Spectrometry

MALDI-TOF mass spectrometry demonstrated that the COS prepared in this study was predominantly composed of low-molecular-weight species ranging from approximately 300 to 2000 Da. The detected ions mainly corresponded to oligomers with degrees of polymerization (DP) of 2–9 (Table 1) [23]. The presence of a regular mass increment of approximately 179.079 Da and 221.089 Da as a monomer between consecutive peaks corresponded to the glucosamine (GlcN) and *N*-acetylglucosamine (GlcNAc) repeating units of the COS backbone, respectively.

The MALDI-TOF MS spectrum indicated dominant peaks at *m*/*z* 344.7934 and 361.7861, which were assigned to the protonated and sodium-adducted forms of a glucosamine dimer ([GlcN_2_ + H]^+^ and [GlcN_2_ + Na]^+^, respectively). This adduct pattern indicates the predominance of low-DP COS, which are primarily composed of deacetylated glucosamine units. Signals were also observed at *m*/*z* 441.0675 and 457.0305 and were tentatively attributed to a GlcNAc-containing dimeric species ([GlcNAc_2_ + NH_4_]^+^ and [GlcNAc_2_ + Na]^+^). These signals suggest the presence of partially acetylated oligomers. The peaks at *m*/*z* 518.7361 and 626.8980 were assigned to trimeric species corresponding to ammonium-adducted glucosamine ([GlcN_3_ + NH_4_]^+^) and protonated *N*-acetylglucosamine oligomers ([GlcNAc_3_ + H]^+^), respectively. Minor peaks were observed in the range of 500–2000 Da, indicating minor amounts of oligomers (DP ≤ 9) (Table 1 and Figures S1 and S2).

### 2.2. ^13^C Nuclear Magnetic Resonance (NMR) Spectroscopy

**Figure 1:**
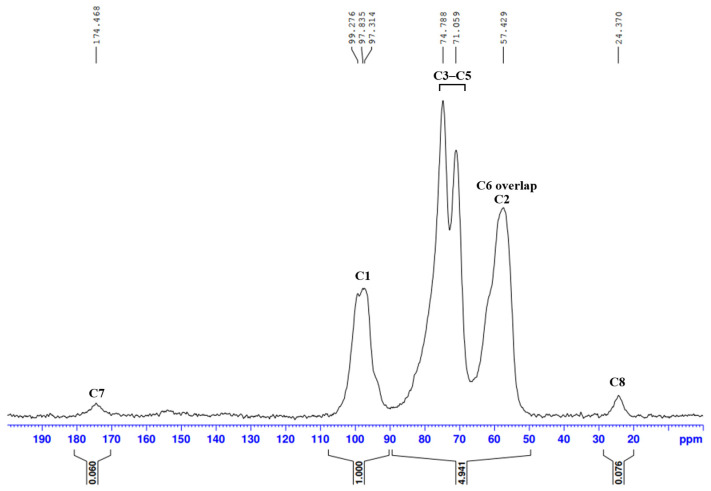
^13^C NMR spectrum (400 MHz) of COS.

The NMR spectrum exhibited characteristic resonances of glucosamine-based oligosaccharide with minor contributions from N-acetylated units (Figure 1). The signals observed at 97–99 ppm were assigned to the anomeric carbons (C1) of the glucopyranose units. Resonances in the range of 71–75 ppm corresponded to ring carbons C3–C5, which are characteristic of hydroxyl-substituted carbon atoms (C–OH). The signal at approximately 57.4 ppm was attributed to C2 bearing an amino group (–CH–NH_2_), indicating the presence of deacetylated glucosamine (GlcN) units. The resonances at ~60–62 ppm were assigned to C6 (–CH_2_OH).

**Figure 2:**
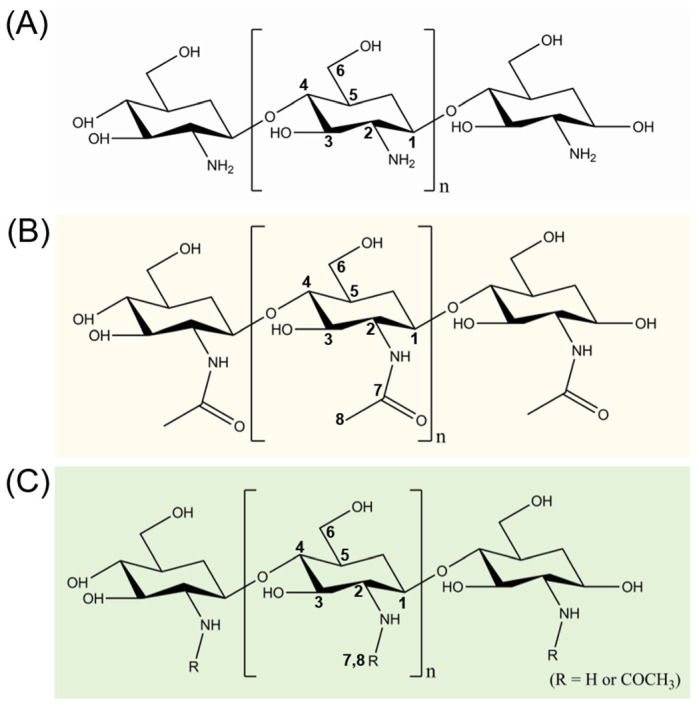
The putative molecular structures of chitosan oligosaccharides (COS) in different forms: (**A**) glucosamine (GlcN)_n_ homooligosaccharides, (**B**) *N*-acetylglucosamine (GlcNAc)_n_ homooligosaccharides, and (**C**) heterooligosaccharides (GlcNAc-GlcN)_n_, where *n* ≤ 9.

The weak signals at approximately 174.5 ppm and 24.4 ppm were assigned to the C7 carbonyl carbon (C=O) and C8 methyl carbon (CH_3_) of the N-acetyl group, respectively, indicating the presence of residual *N*-acetylglucosamine (GlcNAc) units. The NMR spectrum also indicated that the degree of deacetylation (DD) of COS was 92.35%. These features indicate partial acetylation and are consistent with the structure of low-molecular-weight COS (Figure 2) [24,25].

### 2.3. Effect on 3T3-L1 Preadipocyte Cell Viability

The cytotoxic effects of COS on 3T3-L1 preadipocytes were evaluated using the MTT assay and a wide concentration range (10–2560 µg/mL) over 7 days of treatment. COS treatment maintained cell viability for more than 90% relative to vehicle controls at concentrations of up to 1280 µg/mL for all incubation times at days 1, 3, 5, and 7. Even at the highest COS concentration of 2560 µg/mL, cell viability remained consistently high at ~80–85% from day 1 to day 7, indicating minimal cytotoxicity at supraphysiological doses. 

**Figure 3:**
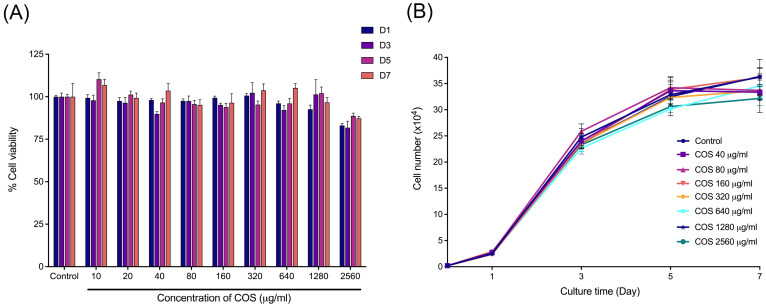
The effects of COS on 3T3-L1 preadipocyte cell viability. (**A**) The viability of 3T3-L1 cells was measured by an MTT assay under the treatments of different concentrations of COS (10–2560 µg/mL) at days 1, 3, 5, and 7. (**B**) Viable cell numbers of 3T3-L1 following COS treatment (40–2560 µg/mL) were evaluated using the trypan blue exclusion assay at days 1, 3, 5, and 7. Each data point is presented as mean ± SEM of four independent experiments. Data were analyzed using one-way ANOVA followed by Tukey’s multiple comparisons test to determine statistically significant differences among groups.

Statistical analysis indicated no significant differences (*p* > 0.05) in cell viability between COS-treated groups (10–2560 µg/mL) and vehicle controls at all time points (Figure 3A). COS treatment with concentrations of up to 2560 µg/mL did not reduce cell viability according to the trypan blue exclusion assay (Figure 3B). Based on these findings, COS concentrations of up to 1280 µg/mL were selected for subsequent adipogenic differentiation experiments as they did not significantly affect either cellular metabolic activity or viable cell numbers.

### 2.4. Effects on Adipogenic Differentiation and Intracellular Lipid Accumulation in Preadipocytes

COS treatment potentially inhibited the adipogenic differentiation of 3T3-L1 preadipocytes in a concentration-dependent manner. 3T3-L1 preadipocytes were induced to differentiate using a standard adipogenic medium supplemented with COS at concentrations of 320, 640, or 1280 μg/mL for 12 days. Undifferentiated controls (Undiff) showed hardly any Oil Red O-stained cells.

**Figure 4:**
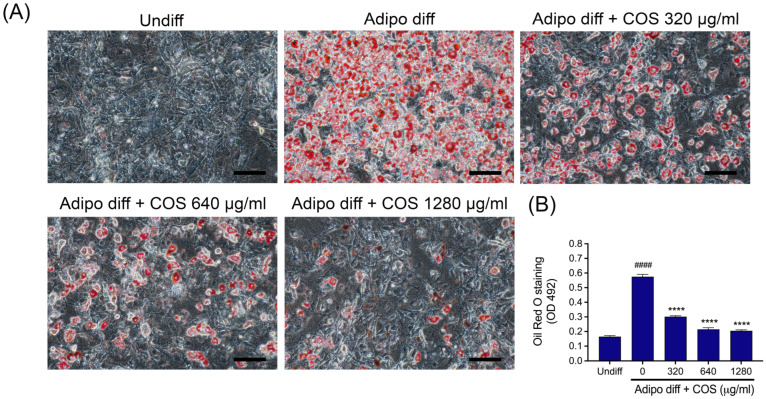
Preventive effect of COS on the adipogenic differentiation of 3T3-L1 cells at concentrations of 320, 640, and 1280 μg/mL over 12 days. (**A**) Representative images of Oil Red O staining, showing intracellular lipid droplets in red of undifferentiated cells (Undiff), adipogenic differentiation control (Adipo diff), and COS-treated differentiated cells at the indicated concentrations, observed at 20× magnification; scale bar = 100 μm. (**B**) The quantification of Oil Red O absorbance in 3T3-L1 cells, as shown in (**A**). Data are presented as mean ± SEM from four independent experiments. #### *p* < 0.0001, adipogenic differentiation control (Adipo) compared with undifferentiated control (Undiff); **** *p* < 0.0001, COS-treated groups compared with adipogenic differentiation control (Adipo diff).

Differentiated controls (Adipo diff) showed the intense red-orange staining of neutral triglycerides and lipid droplets in nearly 95% of cells, indicating substantial intracellular lipid accumulation (Figure 4A). However, COS induced a dose-dependent reduction of 3T3-L1 preadipocytes with red staining to roughly 35% of cells for COS treatment at 320 μg/mL, 25% of cells for 640 μg/mL, and around 15% of cells for 1280 μg/mL (Figure 4A). Quantitative spectrophotometric analysis of extracted Oil Red O at 492 nm indicated that COS effectively inhibited 3T3-L1 adipogenic differentiation and neutral lipid accumulation in a concentration-dependent manner, with significant differences observed at 320, 640, and 1280 μg/mL. These results illustrate the therapeutic potential of COS in combatting obesity-related adipose tissue expansion (Figure 4B).

### 2.5. Effect on Adipocyte Lipid Storage, Lipolysis, and Insulin-Stimulated Glucose Uptake

**Figure 5:**
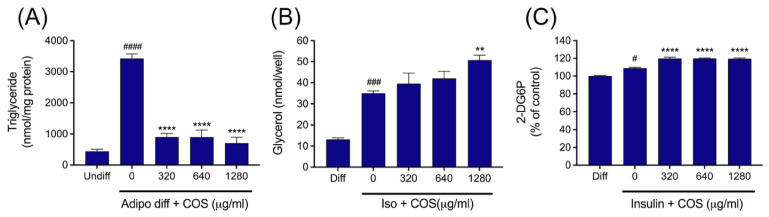
Effects of COS at various concentrations on triglyceride content during adipogenic differentiation (**A**), lipolysis as measured by glycerol release in fully differentiated 3T3-L1 adipocytes (**B**), and glucose uptake measured by 2-deoxyglucose-6-phosphate (2-DG6P) accumulation (**C**) in fully differentiated 3T3-L1 adipocytes. For panels (**B**,**C**), fully differentiated adipocytes were treated with COS after completion of adipogenic differentiation. Differentiated adipocyte controls (Adipo diff) were stimulated with isoproterenol (Iso) and insulin, respectively, as positive controls. Data are presented as mean ± SEM from four independent experiments. # *p* < 0.05, ### *p* < 0.001, #### *p* < 0.0001, adipogenic differentiation control (Adipo diff) compared with undifferentiated control (Undiff); ** *p* < 0.01, **** *p* < 0.0001, COS-treated groups compared with adipogenic differentiation control (Adipo diff).

Intracellular triglyceride content, isoproterenol (Iso)-stimulated lipolysis, and insulin-mediated glucose uptake were evaluated in differentiated mature 3T3-L1 adipocytes treated with COS at 320, 640, and 1280 µg/mL. The intracellular triglyceride content was significantly elevated in the adipogenic differentiation control (Adipo diff) compared with the undifferentiated control (Undiff) (*p* < 0.0001). COS treatment resulted in a significant dose-dependent reduction in triglyceride accumulation, decreasing from 3300 nmol/mg protein in the Adipo diff control to 900, 910, and 800 nmol/mg protein (equivalent to 27.27%, 27.57%, and 24.24% of the Adipo diff control) at 320, 640, and 1280 µg/mL, respectively (Figure 5A).

Iso-stimulated lipolysis, assessed by extracellular glycerol release, was increased by COS co-treatment. The basal glycerol release was 12 nmol/well, while isoproterenol (Iso) stimulation alone yielded 35 ± 3 nmol/well. Co-treatment with isoproterenol (Iso) and COS at 320, 640, and 1280 µg/mL increased glycerol release to 40 ± 4, 42 ± 3, and 50 ± 4 nmol/well, respectively. A statistically significant increase in glycerol release was observed only at 1280 µg/mL compared with the isoproterenol (Iso)-alone group (Figure 5B).

The effect of COS on insulin-mediated glucose uptake was evaluated by measuring intracellular 2-deoxyglucose-6-phosphate (2-DG6P) accumulation. Insulin stimulation alone, serving as the positive control, was normalized to 100 ± 5%. Compared with the insulin-alone control, COS at all tested concentrations, in combination with insulin, significantly increased 2-DG6P accumulation to 120% at 320, 640, and 1280 µg/mL, respectively (Figure 5C).

### 2.6. Inhibition of Adipogenic Transcription Factors in Adipocytes

To examine the effect of COS on key adipogenic transcription factors at the protein level, 3T3-L1 preadipocytes were induced to differentiate in the presence of COS at 320, 640, and 1280 µg/mL. The protein expression of PPARγ, C/EBPα, and β-actin was then assessed by Western blot analysis. Undifferentiated control cells (Undiff) showed a minimal expression of both transcription factors, whereas the adipogenic differentiation control (Adipo diff) exhibited a strong expression of PPARγ and C/EBPα, which is consistent with the characteristics of mature adipocytes.

**Figure 6:**
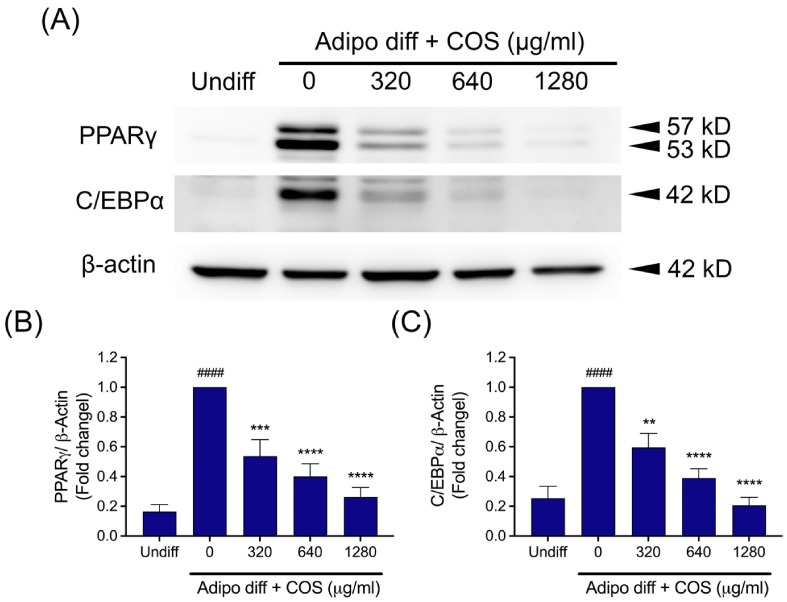
Effects of COS on adipogenic differentiation markers in 3T3-L1 cells. (**A**) Representative Western blot images of PPARγ (~57 and ~53 kDa), C/EBPα (~42 kDa), and β-actin (~42 kDa) in undifferentiated control (Undiff), adipogenic differentiation control (Adipo diff), and COS-treated groups at 320, 640, and 1280 µg/mL. (**B**,**C**) Quantification of relative protein expression levels of PPARγ (**B**) and C/EBPα (**C**), normalized to β-actin. Data are presented as mean ± SEM from six independent experiments. #### *p* < 0.0001, adipogenic differentiation control (Adipo diff) compared with undifferentiated control (Undiff); ** *p* < 0.01, *** *p* < 0.001, **** *p* < 0.0001, COS-treated groups compared with adipogenic differentiation control (Adipo diff).

COS treatment resulted in significant dose-dependent reductions in both PPARγ and C/EBPα expression compared with the Adipo diff control (Figure 6A and Figure S3). Compared with this control, the densitometric quantification of band intensities normalized to β-actin indicated the significant downregulation of PPARγ protein levels to 0.5-, 0.4-, and 0.3-fold at 320, 640, and 1280 µg/mL, respectively (Figure 6B). Similarly, C/EBPα protein levels were significantly reduced to 0.6-, 0.4-, and 0.2-fold at the corresponding concentrations (Figure 6C). These findings demonstrate that COS effectively suppresses the expression of the key adipogenic transcription factors PPARγ and C/EBPα in a dose-dependent manner at the protein level.

### 2.7. Differentially Expressed Genes (DEGs) Under Treatment

DEG analysis was performed by comparing the transcriptomes of 3T3-L1 preadipocytes treated with adipogenic induction medium (control) and COS at 1280 μg/mL in adipogenic induction medium. Each transcriptome generated an average of 6.62 gigabytes of raw reads, and a total of 15,930 genes were detected across all conditions. The average genome mapping rate was 97.99%, and the average gene alignment rate was 75.05%. The Pearson correlation coefficients between biological replicates exceeded 0.989 (Figure S4), indicating high reproducibility.

**Figure 7:**
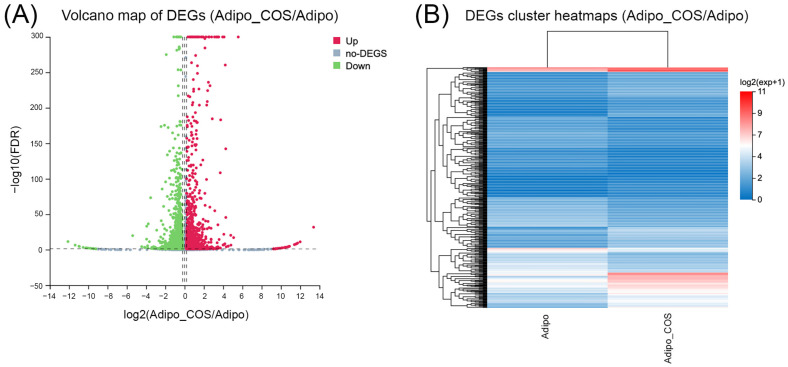
Identification and functional analysis of differentially expressed genes (DEGs) in adipogenic differentiation control (Adipo) compared with COS-treated 3T3-L1 adipocytes. (**A**) Volcano plot of DEGs analyzed by DESeq2 (log_2_ fold change ≥ 0.2; adjusted *p*-value [Qvalue] ≤ 0.05); red dots represent significantly upregulated DEGs, green dots represent significantly downregulated DEGs, and gray dots represent genes that did not exhibit statistically significant differential expression. (**B**) Heatmap illustrating expression profile of DEGs across all samples, standardized by log (expression + 1). Each row represents an individual gene and each column represents a sample; color intensity indicates relative expression level, with blue indicating low expression and red indicating high expression.

The samples’ gene expression distributions and density profiles were consistent with each other (Figures S5 and S6). The comparison between the two groups identified 12,723 DEGs, of which 4461 were upregulated, and 6161 were downregulated (Figure 7A, Supplementary Table S1). The expression heatmap of DEGs clearly demonstrated dose-dependent transcriptional reprogramming by COS, with progressive divergence from control adipogenic profiles (Figure 7B).

### 2.8. Gene Ontology (GO) Enrichment Analysis

The most enriched GO terms were determined in the categories of cellular components (CCs), molecular functions (MFs), and biological processes (BPs). The results highlighted processes that are central to adipocyte biology, including lipid metabolism, cellular signaling, differentiation, and energy homeostasis (Figure S7). The most enriched BP terms (blue bars) captured the multifaceted regulatory landscape of adipocyte differentiation, metabolism, signaling, and the positive regulation of gene expression (master adipogenic control via PPARγ/C/EBPα) and cell differentiation (adipocyte lineage commitment).

The most significantly enriched CC terms (orange bars) spanned subcellular architectures of adipocytes that are essential for lipid handling and prominently featured the cytoplasm and cytosol (primary sites of metabolic enzymes and AMPK signaling), the nucleus and nucleoplasm (transcriptional hubs that regulate PPARγ/C/EBPα), integral components of the membrane (critical for GLUT4 translocation and fatty acid transporters like *Slc27a1*), mitochondrion (β-oxidation via *Cpt1c* upregulation), the cytoskeleton and cell projection (adipocyte remodeling/matrix interactions), and the endoplasmic reticulum and Golgi apparatus (lipid droplet biogenesis/adipokine secretion). The most significantly enriched MF terms (light blue bars) underscored enzymatic, binding, and regulatory activities that are pivotal to adipocyte transcriptional control, RNA polymerase II-specific, and chromatin binding (adipogenic regulators like PPARγ, C/EBPα), protein serine/threonine kinase activity (PI3K-Akt, AMPK cascades), hydrolase activity, and nucleic acid binding. The GO analysis indicates that COS reprograms adipocyte function processes toward anti-adipogenic lipid mobilization at multiple organizational levels (organellar, enzymatic, and process levels) (Figure S7, Supplementary Table S2–S4).

### 2.9. KEGG Pathway Analysis Under COS Treatment

**Figure 8:**
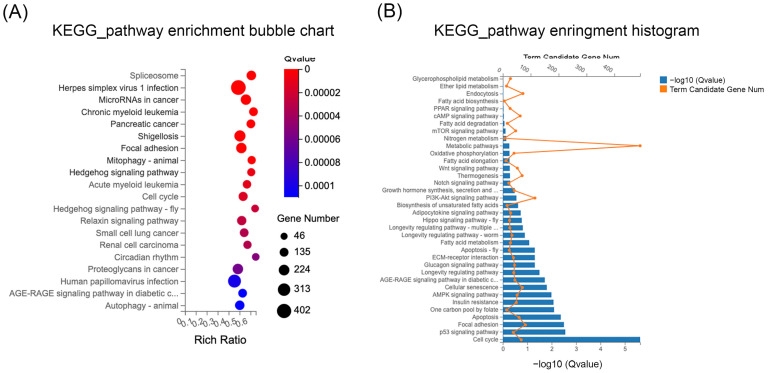
KEGG pathway enrichment analysis of differentially expressed genes (DEGs) in COS-treated 3T3-L1 adipocytes compared with the adipogenic differentiation control (Adipo). (**A**) Bubble chart displaying the top enriched KEGG pathways; bubble size represents number of DEGs enriched in each pathway and bubble color indicates adjusted *p*-value (Q value). (**B**) Enrichment histogram illustrates adipocyte-relevant pathways pertaining to lipid homeostasis and adipocyte function; blue bars represent the −log_10_ (Q value) of each enriched pathway (bottom *x*-axis), and orange line with markers indicates term candidate gene number per pathway (top *x*-axis). A complete list of all enriched KEGG pathways with corresponding gene counts and statistical values is provided in Supplementary Table S5.

According to the KEGG (Kyoto Encyclopedia of Genes and Genomes) analysis, the top 20 most significantly enriched pathways of differentially expressed genes (DEGs) primarily encompassed those related to cancer, the herpes simplex virus 1 infection, and microRNAs in cancer, among others (Figure 8A, Supplementary Table S5). Although predominantly featuring generic cellular-stress and oncogenic pathways, these enrichments reveal COS’s broad impact on fundamental cellular processes, including autophagy, ECM interactions, MAPK signaling, and PI3K-Akt signaling, which intersect with adipogenic regulation. Critically, adipocyte-related pathways demonstrate COS’s specific mechanistic action despite not ranking in the statistical top 20. These include PPAR signaling, AMPK signaling, insulin signaling, fatty acid degradation, and adipocytokine signaling, which were identified through the targeted pathway analysis of biologically relevant DEGs (Figure 8A, Supplementary Table S5).

We also investigated adipocyte-related pathways based on biological significance, −log10(Q value), and term candidate gene number from the enrichment histogram. Multiple pathways that are directly related to adipocyte function and lipid homeostasis appeared at various positions. These included the AMPK signaling pathway (rank 32), FoxO signaling pathway (rank 33), insulin signaling pathway (rank 57), PI3K-Akt signaling pathway (rank 61), MAPK signaling pathway (rank 55), adipocytokine signaling pathway (rank 99), PPAR signaling pathway (rank 152), glucagon signaling pathway (rank 171), non-alcoholic fatty liver disease (rank 124), insulin resistance (rank 146), peroxisome (rank 139), fatty acid metabolism (rank 142), fatty acid biosynthesis (rank 193), the regulation of lipolysis in adipocytes (rank 195), and thermogenesis (rank 150). These biologically critical pathways were interspersed among generic cellular processes and demonstrate COS’s targeted modulation of energy sensing (AMPK), transcriptional regulation (PPAR, FoxO), glucose/lipid handling (insulin, PI3K-Akt), endocrine signaling (adipocytokine, glucagon), and lipid catabolism (peroxisome, lipolysis). These results provide mechanistic validation of the anti-adipogenic effects of COS despite variable statistical rankings (Figure 8B, Supplementary Table S5).

### 2.10. Adipogenic Differentiation Gene Expression in Response to COS Treatment

Multiple signaling pathways facilitate the COS-induced inhibition of adipogenesis in 3T3-L1 preadipocytes. On day 12 of adipogenic differentiation, transcriptional alterations between the control adipogenic induction medium and COS-treated groups (1280 μg/mL) were significantly linked to five principal signaling pathways: The PPAR signaling pathway, PI3K-Akt signaling pathway, insulin signaling pathway, AMPK signaling pathway, and fatty acid degradation pathway. The DEGs with FDR q-values less than 0.05, and log2 fold change > 0.2 were considered significant, as detailed in Table 2, Table 3, Table 4 and Table 5. Significant findings reveal the marked downregulation of PPARγ alongside the activation of catabolic regulators (*Acox2*, *Cpt1c*) in PPAR signaling, substantial AMPK activation (*Prkaa2*, *Sirt1*) in conjunction with PI3K inhibition (*Pik3cd*, *Pik3r1*), and the disruption of insulin signaling (*Phka1*, *Socs2*). These results demonstrate the complex mechanisms involved in COS inhibiting lipid accumulation and adipocyte maturation.

Differential expression analysis of COS-treated 3T3-L1 adipocytes indicated a significant modulation of genes associated with PPAR signaling and fatty acid metabolism pathways (Table 2), which is consistent with the COS-mediated suppression of adipogenesis and lipid accumulation. In the PPAR signaling pathway, COS treatment markedly downregulated *Pparg* (peroxisome proliferator-activated receptor gamma, −0.46), which is the master regulator of adipocyte differentiation and lipid storage. It also reduced the expression of lipid droplet-associated proteins *Plin1* (perilipin 1, −0.69) and *Plin4* (perilipin 4, 0.37), which coat lipid droplets and regulate lipolysis.

Similarly, *Fabp5* (fatty acid-binding protein 5, −0.39) and *Fads2* (fatty acid desaturase 2, −0.35) showed decreased expression, which reflects impaired fatty acid uptake and lipid modification. Conversely, the upregulation of *Rxrb* (retinoid X receptor beta, 2.51) suggests the compensatory activation of PPAR heterodimer partners that may promote alternative metabolic programs. With regard to fatty acid degradation, the COS induced upregulation of key catabolic enzymes, including *Acox2* (acyl-CoA oxidase 2, 10.10), *Cpt1c* (carnitine palmitoyltransferase 1C, 1.45), *Hmgcs2* (3-hydroxy-3-methylglutaryl-CoA synthase 2, 0.93), and *Acox3* (acyl-CoA oxidase 3, 0.56), which is indicative of increased peroxisomal β-oxidation and mitochondrial fatty acid transport.

The upregulation of *Nr1h3* (liver X receptor α, 2.23) also supports cholesterol efflux, lipolysis, and fatty acid oxidation. However, there was also suppression of lipid synthesis regulators *Aqp7* (aquaporin 7, −3.17), *AcsL3* (acyl-CoA synthetase long-chain family member 3, −2.23), and *Slc27a1* (solute carrier family 27 member 1, −1.41), which is a glycerol channel linked to lipolysis. This suppression aligns with reduced de novo lipogenesis. AMPK-related genes such as *Pdpk1* (3-phosphoinositide dependent protein kinase 1, 1.73) and *Ilk* (integrin-linked kinase, −0.71) showed mixed regulation, which potentially reflects AMPK activation inhibiting anabolic processes while promoting energy homeostasis.

The AMPK signaling pathway and the suppression of the insulin and PI3K-Akt pathways (Table 3) establish a mechanistic basis for COS-mediated metabolic reprogramming. In the AMPK pathway, COS markedly upregulated core components, including *Tsc1* (tuberous sclerosis complex 1, 12.73), *Prkaa2* (Protein kinase, AMP-activated, catalytic α2, 11.81), *Prkag2* (protein kinase AMP-activated non-catalytic γ2, 1.98), *Sirt1* (sirtuin 1, 9.42), and *Cab39* (calcium-binding protein 39, 1.17). This result indicates robust AMPK activation that promotes energy homeostasis, autophagy, *Ulk1* (Unc-51 like autophagy activating kinase 1, 1.03), and catabolic metabolism. The upregulation of *Creb3l4* (cyclic AMP-responsive element-binding protein 3-like protein 4, 9.45) further supports the occurrence of the AMPK-dependent transcriptional regulation of stress responses.

Conversely, the insulin and PI3K-Akt signaling pathways were prominently downregulated, as evidenced by the strong suppression of *Igf1* (insulin-like growth factor 1, −11.93), *Ppp2r5c* (PP2A regulatory subunit, −9.70), *Pik3cd* (PI3K catalytic subunit δ, −2.63), *Pik3r3* (PI3K regulatory subunit γ, −0.59), and *Pik3r1* (PI3K regulatory subunit 1, −0.45), which collectively attenuate PI3K-Akt signaling and insulin responsiveness. The inhibition of insulin-driven lipogenesis is reinforced by the downregulation of *Srebf1* (sterol regulatory element-binding transcription factor 1, −2.20), a key lipogenic transcription factor downstream of Akt, alongside *Lipe* (lipase E, −0.41). The paradoxical upregulation of *Akt2* (14.28) and *Akt1s1* (−0.54) suggests context-specific Akt isoform regulation, which may potentially reflect feedback mechanisms.

COS-treated 3T3-L1 adipocytes demonstrated a substantial remodeling of the PI3K-Akt signaling pathway, which was characterized by the upregulation of pro-apoptotic regulators and the downregulation of growth factor signaling components (Table 4). Notably, COS induced the dramatic upregulation of *Bcl2l11* (Bcl-2-like protein 11/Bim, 11.91), a pro-apoptotic BH3-only protein that antagonizes anti-apoptotic Bcl-2 family members and promotes cell death pathways, alongside *Lpar1* (lysophosphatidic acid receptor 1, 11.88), which can modulate PI3K-Akt via G-protein coupled receptor signaling. The COS activation of intrinsic apoptotic programs counteracts PI3K-Akt-mediated survival signaling.

Conversely, COS profoundly suppressed multiple upstream activators and effectors of the PI3K-Akt cascade, including *Pkn1* (Protein kinase N1, −14.08), a PKC-related kinase that scaffolds Akt activation; *Ywhaz* (tyrosine 3-monooxygenase/tryptophan 5-monooxygenase activation protein zeta, −12.50), which stabilizes phosphorylated Akt and promotes cell survival; *Fgfr3* (fibroblast growth factor receptor 3, −11.29); *Ptk2* (protein tyrosine kinase 2, −11.04); *Egf* (epidermal growth factor, −10.93); *Bdnf* (brain-derived neurotrophic factor, −9.23); and *Vegfa* (vascular endothelial growth factor A, −0.89). These growth factors and receptor tyrosine kinases typically trigger PI3K activation through receptor tyrosine phosphorylation, which leads to PIP3 production, Akt membrane recruitment, and downstream anabolic signaling. The coordinated downregulation disrupts this axis, which leads to the inhibition of insulin/IGF-stimulated glucose uptake, protein synthesis, and cell proliferation while favoring catabolic processes.

COS-treated 3T3-L1 adipocytes exhibited the differential expression of genes that are connected to the insulin signaling pathway, with the downregulation of genes associated with glycogen metabolism and MAPK/ERK signaling (Table 5). COS treatment induced the upregulation of *Mapk8* (mitogen-activated protein kinase 8, 0.56) and *Mapk1* (mitogen-activated protein kinase 1, 0.30), which suggests the activation of JNK/ERK stress signaling branches that antagonize insulin action and promote catabolism. In contrast, profound downregulation was observed for *Phka1* (phosphorylase kinase regulatory subunit α1, −11.89), a key insulin-responsive regulator of glycogenolysis; *Socs2* (suppressor of cytokine signaling 2, −9.64), which modulates insulin/IGF-1 signaling feedback; *Mapk9* (mitogen-activated protein kinase kinase 2, −5.07); *Exoc7* (exocyst complex component 7, −3.42), which is essential for GLUT4 vesicle trafficking; *Map2k2* (mitogen-activated protein kinase kinase 2, −1.44); and *Hk1* (hexokinase 1, −0.87), the rate-limiting enzyme for glucose phosphorylation.

## 3. Discussion

COS have attracted considerable attention as promising nutraceutical candidates for obesity and metabolic syndrome, yet their direct molecular actions in adipocytes have not been completely defined [26]. Although studies have provided valuable insights into the anti-adipogenic potential of COS, the integrated transcriptional response to low-molecular-weight COS fractions in adipocytes has not been fully characterized. In the present study, a low-molecular-weight COS preparation (DP 2–9, highly deacetylated) was structurally characterized and was shown to exert potent anti-adipogenic and metabolic effects in 3T3-L1 preadipocytes, which resulted in the marked suppression of adipocyte differentiation, intracellular triglyceride accumulation, and lipogenic transcription factors while preserving cell viability [14,15].

These cellular responses were accompanied by reduced triglyceride storage due to coordinated metabolic reprogramming, increased β adrenergic lipolysis, and improved insulin-stimulated glucose uptake. These effects arose from the comprehensive transcriptomic remodeling of PPAR, AMPK, PI3K Akt, insulin, and fatty acid metabolism pathways. These data indicate that COS do not function merely as passive fat-binding polymers, as suggested in early clinical work. Instead, they function as active regulators of adipocyte fate and energy homeostasis. This mechanistic profile of COS supports reports of COS mediating reductions in body weight, adiposity, and hepatosteatosis in vivo. Therefore, this study reconciles divergent findings from earlier 3T3-L1 studies that COS play significant roles in either anti- or pro-adipogenic effects depending on the molecular structure and context.

Our MALDI-TOF data indicated that the COS preparation used in this study is highly rich in low-molecular-weight oligomers (DP 2–9; major size at 300–400 Da), with a predominance of GlcN dimers and other short chains. This profile is broadly consistent with enzymatically generated COS preparations that have shown robust anti-obesity and metabolic benefits in rodent and human studies [7,22,27]. In anti-adipogenic models, low-DP fractions generally show stronger bioactivity than chitosan with higher molecular weight, which likely results from shorter chains exhibiting higher aqueous solubility, surface charge density, and accessibility to cell-surface receptors and transporters [5,22]. Compared with larger COS, GO2KA1, and other commercial COS, which often have a broader distribution extending up to 5–10 kDa, our preparation is shifted toward very low-DP species, which underly the relatively strong inhibition of adipogenesis and transcriptional reprogramming observed in this study and others [7,27,28]. Thus, the MALDI-defined composition supports the notion that the precise control of DP is a critical determinant of COS bioactivity [12].

^13^C NMR analysis corroborated the MALDI-TOF findings and showed dominant resonances corresponding to GlcN units with only minor signals from N-acetyl groups, indicating a highly deacetylated backbone [24]. Earlier structure–activity studies have suggested that a high degree of deacetylation increases the cationic character of COS and promotes electrostatic interactions with negatively charged membranes, receptors, and nucleic acids [29]. These effects improve the anti-obesity efficacy in vivo [5,7,22]. At the same time, residual GlcNAc units generally help to preserve chain flexibility and aqueous solubility [23,30], which can support systemic distribution [31].

The combined MS and NMR characterization places our COS in a structural window that is associated with optimal anti-adipogenic and anti-diabetic activities and provides a solid basis for interpreting their downstream biological effects [7,22]. This structural information allows us to infer the chain length, charge density, and hydrogen-bonding networks of the COS molecules. Low-molecular-weight oligosaccharides show higher aqueous solubility, membrane permeability, and cellular uptake compared to those with high molecular weight [31,32], Thus, the predominance of dimeric species (DP2) in our preparation is likely to be critical for achieving efficient intracellular bioactivity.

Larger chitosan polymers are largely confined to extracellular or membrane-associated actions [33,34]. In contrast, precisely defined low-molecular-weight COS are more likely to traverse cell membranes and directly engage intracellular signaling pathways. This is evidenced by their high apparent permeability coefficients across epithelial cell monolayers [35] and their capacity to activate intracellular AMPK signaling in epithelial cells [12].

COS maintained more than 90% viability in 3T3-L1 preadipocytes with treatment at up to 640 μg/mL and only modestly reduced viability with treatment at 2560 μg/mL during 7-day exposure. This indicates that the anti-adipogenic effects are not confounded by overt cytotoxicity. This is consistent with 3T3-L1 studies in which COS at 0.5–4 mg/mL altered differentiation and lipid accumulation without major effects on cell survival. Proteomic analyses showed widespread changes in metabolic enzymes and cytoskeletal proteins in COS-treated adipocytes without the activation of classical apoptosis markers, such as PPARγ and C/EBPα, which supports a primarily regulatory rather than cytotoxic mode of action [16]. Many phytochemicals and synthetic PPAR modulators exhibit narrow therapeutic windows due to pro-apoptotic effects near their effective concentrations [36,37]. In contrast, COS appeared to reprogram adipocyte metabolism in a broad safety margin, which is consistent with their favorable tolerability profile in clinical trials for obesity and metabolic syndrome [7,26,27].

COS markedly suppressed 3T3-L1 adipogenesis, which was evidenced by a reduction in Oil Red O positive cells from ~95% in differentiated controls to 15–35% in COS-treated cultures, as well as a 73–76% decrease in triglyceride content. These effects are comparable to or stronger than those reported for other low-molecular-weight COS preparations, which typically reduce lipid accumulation by 40–70% at similar doses of COS or sulfated chitosan oligomer and inhibit the expression of adipogenic markers such as PPARγ, C/EBPα, FABP4, and GPDH. Furthermore, they interfere with the cytoskeletal reorganization required for terminal differentiation [5,38]. Our data are consistent with this paradigm but demonstrate more a profound inhibition of morphological maturation, which likely reflects the higher proportion of very low-DP and highly deacetylated chains.

Interestingly, not all COS behave purely as anti-adipogenic agents. GO2KA1 has been reported to increase adipocyte differentiation while improving insulin sensitivity, which highlights how differences in DP distribution, dosing regimen, and culture conditions can shift COS from PPARγ antagonists to partial agonists [39]. Therefore, the strong blockade of differentiation observed emphasizes the importance of precise structural characterization when comparing COS studies.

In mature 3T3-L1 adipocytes, COS treatment decreased intracellular triglycerides by ~75%, modestly increased isoproterenol stimulated lipolysis, and increased insulin-stimulated glucose uptake by ~20%. Studies in vivo have shown that COS ameliorate obesity and hepatic steatosis, reduce serum triglycerides and cholesterol, and increase insulin sensitivity in high-fat-diet models and clinical settings [15,40]. At the cellular level, GO2KA1 has been reported to increase glucose uptake in 3T3-L1 cells through the upregulation of GLUT4 and adiponectin in a PPARγ-dependent manner. Other COS formulations reduce GLUT4 and lipogenic enzymes and shift adipocytes toward a more oxidative phenotype [5,16,28,39].

The concurrent reduction in intracellular lipid storage alongside increased insulin-stimulated glucose uptake in COS-treated 3T3-L1 adipocytes raises the hypothesis that COS may contribute to a metabolic shift supporting reduced lipid accumulation and improved insulin-mediated glucose utilization. In support of this hypothesis, transcriptomic analysis indicated the coordinated upregulation of *Cab39* (MO25), *Prkaa2* (AMPKα2), and *Prkag2* (AMPKγ2), components of the LKB1–STRAD–MO25/AMPK axis, required for GLUT4 translocation via TBC1D4/AS160 phosphorylation [40,41,42]. The concurrent downregulation of Ppp2r5c, a phosphatase that inactivates AMPK at Thr172, further supports sustained AMPK activation [43]. Although upstream PI3K components were transcriptionally suppressed, the isoform-specific upregulation of Akt2 is consistent with the selective preservation of insulin-stimulated glucose uptake as Akt2 activation alone is sufficient to phosphorylate AS160 and stimulate GLUT4 translocation independently of PI3K in 3T3-L1 adipocytes [44,45]. Both AMPK and Akt2 converge on TBC1D4/AS160 [41], which provides a dual mechanistic basis that aligns PI3K suppression with the functionally observed increase in glucose uptake.

Protein profile data showing the dose-dependent suppression of PPARγ and C/EBPα provides direct molecular support for the observed anti-adipogenic effects. Similar reductions in PPARγ and its downstream targets (FABP4, GPDH, GLUT4) have been reported in 3T3 L1 cells treated with COS or sulfated chitosan oligomer, as well as in the adipose tissue of COS-supplemented obese rats [5,7,16,38]. In contrast, other low-molecular-weight COS increase PPARγ expression and adiponectin in adipocytes while simultaneously exerting anti-diabetic effects through the inhibition of intestinal α-glucosidase and glucose transporters [39]. These contrasting outcomes underscore that the molecular weight, structural composition, and experimental context of COS strongly influence the PPARγ signaling outcomes. Our transcriptomic data extend the protein-level findings by demonstrating that *Pparg* and multiple PPARγ target genes (*Plin1*, *Acsl3*, *Fabp5*, *Fads2*) are significantly downregulated, whereas genes involved in fatty-acid oxidation (*Acox2*, *Cpt1c*, *Hmgcs2*) are upregulated. This is consistent with reported COS-induced shifts toward fatty acid oxidation in metabolic tissues [5,22]. These results support a model in which the present COS preparation functions predominantly as a PPARγ antagonist in adipocytes that favor a catabolic low-lipid state.

RNA-seq analysis identified 12,723 DEGs between COS-treated and control adipocytes. These represent a whole range of transcriptional changes induced by COS during transition from proliferating preadipocytes to mature adipocytes. Although the differentiation processes of these cells are typically associated with the upregulation of lipogenic and extracellular matrix genes and the downregulation of cell cycle genes, COS treatment produced an inverse signature characterized by the suppression of adipogenic and growth factor genes and the upregulation of catabolic, stress response, and autophagy-related genes. This pattern is consistent with in vivo reports that COS reverses the high-fat-diet-induced expression of lipogenic, inflammatory, and ER stress genes in liver and adipose tissues while increasing the expression of genes involved in fatty acid oxidation and antioxidant defense [5,22,46,47,48].

GO enrichment analysis indicated that COS impact multiple levels of adipocyte biology. At the biological process level, terms related to transcriptional regulation, cell differentiation, cell cycle, apoptosis, and lipid metabolism were highly enriched, which aligns with previous data showing that COS can alter leptin gene methylation and transcription, attenuate inflammatory signaling, and modulate cell cycle regulators in adipose tissue [22,26,49]. Cellular component enrichment in the cytoplasm, mitochondria, peroxisome, endoplasmic reticulum, plasma membrane, and lipid-related organelles mirror proteomic findings that COS alter enzymes and structural proteins associated with β-oxidation, glycolysis, and lipid droplet dynamics [5,50]. Molecular function categories such as protein binding, kinase activity, oxidoreductase activity, and DNA-binding transcription factor activity further support the notion that COS simultaneously target signaling kinases, metabolic enzymes, and transcriptional regulators and provide a mechanistic basis for the broad range of phenotypic changes observed [22].

The top 20 KEGG pathways were dominated by generic processes related to cancer, infection, and RNA processing. However, closer inspection of pathways ranked by −log10(Q) value and candidate gene number indicated the strong enrichment of adipocyte-related networks, including AMPK, PPAR, insulin, PI3K Akt, adipocytokine, fatty acid metabolism, peroxisome, thermogenesis, and the regulation of lipolysis in adipocytes. Similar pathway signatures have been reported in obese rodents that were treated with COS. Improvements in body weight and insulin sensitivity were accompanied by the modulation of AMPK, PPARγ, SREBP 1c, and adipocytokine signaling [19,51]. Our data provide cell-intrinsic evidence that COS directly engage these energy-sensing and lipogenic pathways in adipocytes rather than acting solely via intestinal or hepatic mechanisms, as suggested in an earlier work [27].

Integrative analysis of DEGs in PPAR, AMPK, insulin, and PI3K-Akt pathways showed that COS orchestrates a coherent network level shift from lipid storage to lipid oxidation and stress resilient energy homeostasis. The upregulation of *Acox2*, *Acox3*, *Cpt1c*, *Hmgcs2*, and *Nr1h3* indicated increased peroxisomal and mitochondrial β oxidation and cholesterol efflux, which occurred in parallel with observations that COS reduce hepatic steatosis and improve serum lipid profiles in animal models [5,52,53]. The concurrent downregulation of *Pparg*, *Plin1*, *Acsl3*, *Slc27a1*, and *Aqp7* reflects the suppression of triglyceride synthesis, fatty acid uptake, and glycerol transport, which is consistent with studies in vitro and in vivo showing the reduced expression of lipogenic genes following COS treatment [5,22,28].

In the AMPK pathway, the strong induction of *Prkaa2*, *Prkag2*, *Sirt1*, *Cab39*, *Ulk1,* and *Tsc1* suggested the robust activation of cellular energy sensing and autophagy. These mechanisms have been implicated in COS-mediated protection against ER stress, oxidative damage, and mitochondrial dysfunction in metabolic tissues [54]. Conversely, the pronounced downregulation of *Igf1*, *Ppp2r5c*, *Pik3cd*, *Pik3r1/3*, *Srebf1*, and *Lipe* indicated the broad attenuation of insulin/PI3K-Akt-driven anabolism [43,55,56,57]. These changes mirrored findings that COS supplementation reduces circulating insulin and IGF-1 levels and downregulates SREBP-1c and ACC1 in liver and adipose tissue, thereby limiting *de novo* lipogenesis [5,19]. The marked upregulation of pro-apoptotic *Bcl2l11* [58] and the downregulation of multiple growth factors and receptors (*FGFR3*, *EGF*, *BDNF*, *Vegfa*) also point to selective elimination or the functional silencing of hypertrophic adipocytes by COS. This perspective is supported by cross-species transcriptomic analyses of adipose remodeling associated with obesity [59,60].

The findings of this study place COS in a growing class of natural compounds that exert multi-target control over the fate and function of adipocytes. By integrating structural characterization with phenotypic, biochemical, and transcriptomic data, the present study demonstrated that a well-defined, low-DP, highly deacetylated COS preparation suppressed adipogenesis, promoted lipid catabolism, and rewired key signaling networks (PPAR, AMPK, insulin, PI3K Akt) in a manner that was highly consistent with its reported anti-obesity and metabolic syndrome benefits in vivo [51,61]. Nevertheless, this study still has limitations. The 3T3-L1 model is a simplified murine in vitro system that does not fully recapitulate the complex pathophysiology of obesity in vivo, including systemic endocrine regulation, inter-organ communication, immune interactions, and whole-body metabolic responses. Thus, future studies should systematically investigate structure–activity relationships more systematically, validate these mechanisms in primary human adipocytes and animal models, evaluate the potential synergism between COS and anti-obesity pharmacotherapies. Pharmacokinetic, dose-optimization, and in vivo studies are also required to determine the biologically relevant and therapeutically achievable concentrations of low-molecular-weight COS [12].

## 4. Materials and Methods

### 4.1. Production of Chitosan and COS

For the extraction and production of COS, crab shells were thoroughly washed with distilled water to remove impurities and dried in a hot-air oven at 70 °C for 6 h. The dried shells were ground into fine powder and subjected to demineralization by treatment with 3 M HCl under continuous stirring at room temperature for 6 h. After acid treatment, the sample was washed with distilled water, followed by absolute ethanol, then dried at 70 °C for 18 h. Deproteinization was performed by treatment with 1 M NaOH at 70 °C for 24 h. The mixture was washed thoroughly with distilled water until a neutral pH was achieved, then washed with absolute ethanol and dried at 70 °C for 18 h. For deacetylation, the obtained chitin was treated with 12.5 M NaOH at 70 °C for 6 h. After incubation, the sample was washed with distilled water to a neutral pH and dried at 70 °C for 18 h to obtain chitosan [62].

COS were prepared by acid hydrolysis of chitosan. Briefly, chitosan powder was dissolved in 2 M HCl and stirred continuously at 70 °C for 2 h. The hydrolyzed chitosan was precipitated by the addition of absolute ethanol and incubated at 4 °C for 18 h. The precipitate was collected by centrifugation, washed with absolute ethanol, and dried in a hot-air oven at 70 °C until constant weight was achieved, to obtain the COS product for further characterization [63].

### 4.2. Characterization and Identification of COS

The molecular mass distribution of COS was analyzed using matrix-assisted laser desorption/ionization time-of-flight mass spectrometry (MALDI-TOF MS) on a SpiralTOF MALDI Imaging-TOF/TOF mass spectrometer (JMS-S3000, JEOL, Tokyo, Japan) operated in positive ion mode. 2,5-Dihydroxybenzoic acid (DHB) was used as the matrix due to its suitability for carbohydrate analysis [64]. Mass spectra were acquired over *m*/*z* ranges, including 300–500 Da and 500–5000 Da. The detected ions were predominantly observed as protonated and sodium-adduct species ([M + H]^+^, [M + NH4]^+^, and [M + Na]^+^) [64]. Data acquisition and spectral processing were performed using the instrument’s proprietary software.

The chemical structure of COS was further characterized by solid-state ^13^C CP/MAS NMR spectroscopy using a 400 MHz Fourier transform NMR spectrometer (AVANCE III HD Ascend 400 WB, Bruker, Billerica, MA, USA). Spectra were acquired with a spectral width of 29,761.9 Hz, 1600 scans, an acquisition time of 0.0344 s, and a relaxation delay of 5.0 s. The receiver gain (RG) was set to 199.18, and the dwell time (DW) was 16.8 μs. Cross-polarization was performed with a contact time of 3.0 ms, and proton decoupling was applied during acquisition. Chemical shifts (δ) were reported in ppm relative to an external reference. The ^13^C NMR spectra were used to identify characteristic resonances corresponding to anomeric carbons, ring carbons, and residual N-acetyl carbonyl groups, thereby confirming the glucosamine-based backbone and partial acetylation of COS [65]. Chitosan oligosaccharide lactate (average mass 5000 Da; CAS No. 148411-57-8, Sigma-Aldrich, St. Louis, MO, USA) was used as a standard compound. The degree of deacetylation (DD) was calculated based on the intensity ratio of the methyl carbon (*I*_CH3_) to ring-carbons (*I*_C1_, *I*_C2_, *I*_C3_, *I*_C4_, *I*_C5_, and *I*_*C*6_) according to the following equation, where *I* represent the signal intensity of C1–C6 and CH_3_ obtained from the NMR spectrum [66].

$$DD=1-\frac{I_{CH3}}{\left(I_{C1}+I_{C2}+I_{C3}+I_{C4}+I_{C5}+I_{C6}\right)/6}$$

### 4.3. 3T3-L1 Preadipocyte Cell Culture and Cell Viability Assay

3T3-L1 preadipocyte cells (ATCC CL-173), derived from mouse embryo, were purchased from the American Type Culture Collection (ATCC, Manassas, VA, USA). Cells were maintained in a preadipocyte growth medium (GM; Dulbecco’s Modified Eagle’s Medium with high glucose, DMEM-HG; Cytiva, Marlborough, MA, USA) supplemented with 10% bovine calf serum (CS; ATCC, Manassas, VA, USA), 100 units/mL penicillin, and 100 µg/mL streptomycin (P/S; Gibco, Thermo Fisher Scientific, Waltham, MA, USA) at 37 °C in a humidified atmosphere of 5% CO_2_/95% air. The medium was routinely replaced every 2–3 days, and cells were subcultured using 0.25% Trypsin-EDTA solution (Gibco, Thermo Fisher Scientific, Waltham, MA, USA) before reaching confluence.

For the cell viability assay, MTT (3-(4, 5-dimethylthiazolyl-2)-2, 5-diphenyltetrazolium bromide) assay, and 3T3-L1 cells were seeded into a 96-well plate at a density of 2 × 10^3^ cells per well in triplicate. After 24 h of incubation, cells were treated with COS at concentrations ranging from 10 to 2560 µg/mL for 1, 3, 5, and 7 days. At each time point, the medium was replaced with 100 µL of MTT solution (0.5 mg/mL; Sigma-Aldrich, Merck KGaA, Darmstadt, Germany) and cells were incubated for 2 h at 37 °C under 5% CO_2_ in a humidified incubator. Subsequently, the supernatants were removed, and the formazan crystals were dissolved in 100 µL of dimethyl sulfoxide (DMSO; Merck KGaA, Darmstadt, Germany). The absorbance in each well was measured by using a microplate reader (Varioskan Flash, Thermo Fisher Scientific, Waltham, MA, USA) at 570 nm with 630 nm as the reference wavelength. Cell viability (%) was calculated and normalized to vehicle control. The experiment was performed with at least four replicates.

Moreover, cell viability was evaluated using the trypan blue exclusion assay to determine the number of viable cells following COS treatment. Briefly, 3T3-L1 cells were trypsinized and seeded into a 24-well plate at a density of 1 × 10^4^ cells per well. After 24 h of incubation, the cells were treated with COS at concentrations ranging from 40 to 2560 µg/mL. On days 1, 3, 5, and 7 following treatments, cells were harvested by trypsinization and stained with trypan blue solution. Viable cells were counted using a hemocytometer. All experiments were performed in four replicates, and the results were expressed as the number of viable cells at each time point. Therefore, COS concentrations up to 1280 µg/mL were selected for subsequent mechanistic studies based on both cellular metabolic activity and viable cell numbers.

### 4.4. Adipogenic Differentiation and COS Treatment

3T3-L1 preadipocytes were used to screen the anti-adipogenic effects of COS. Cells were seeded at 5 × 10^4^ cells per well in 12-well plates and maintained in GM until reaching 100% confluence. Three days post-confluence (designated day 0), adipogenic differentiation was induced by replacing GM with differentiation induction medium (MDI) consisting of DMEM-HG supplemented with 10% fetal bovine serum (FBS), 1% P/S, 1 µM dexamethasone, 0.5 mM 3-isobutyl-1-methylxanthine (IBMX), and 10 µg/mL insulin (Sigma-Aldrich, Merck KGaA, Darmstadt, Germany). On days 3 and 6, the medium was replaced with adipocyte maintenance medium (MM) containing DMEM-HG, 10% FBS, 1% P/S, and 10 µg/mL insulin. From day 9, cells were maintained in GM. Unless otherwise stated, cells were harvested on day 12. To assess the effects on adipogenesis, COS at concentrations of 160, 320, 640, and 1280 µg/mL were added at every medium change throughout the adipogenic differentiation period.

### 4.5. Oil Red O Staining, Extraction and Quantification

Cells were seeded at 5 × 10^4^ cells/well in 12-well plate and differentiated with MDI, MM, and GM in the presence or absence of COS (320, 640 and 1280 µg/mL) as described above. Oil Red O staining was performed on fully differentiated adipocytes at day 12. Cells were washed with phosphate-buffered saline (PBS) and fixed in 10% formalin for 30 min at room temperature. After cells were washed with distilled water (dH_2_O), 60% Isopropanol (Merck KGaA, Darmstadt, Germany) was incubated with the cells for 5 min. A freshly prepared 0.3% (*w*/*v*) Oil Red O working solution (Sigma-Aldrich, Merck KGaA, Darmstadt, Germany), prepared by diluting a 0.5% stock in isopropanol (3:2, *v*/*v* with dH_2_O) and filtered through Whatman No. 1 filter paper, was applied to fixed cells for 30 min at room temperature. Adipocytes were visualized and imaged using an inverted phase contrast microscope (Nikon Eclipse Ti2, Tokyo, Japan). For the quantification of intracellular lipid content, Oil Red O was eluted with 100% isopropanol, and 150 µL of the extracted solution was measured using a microplate reader at an absorbance 492 nm.

### 4.6. Lipolysis Assay

The effect of COS on lipolysis was evaluated in differentiated 3T3-L1 adipocytes using a lipolysis colorimetric assay (ab185433, Abcam, Cambridge, UK) according to the manufacturer’s instructions. Briefly, 3T3-L1 preadipocytes were seeded in 24-well plates at a density of 1 × 10^4^ cells/well and cultured in GM until confluence as described above. Adipogenic differentiation was induced with MDI induction medium on day 0, followed by MM on days 3 and 6, and GM on day 9. On day 12, following complete differentiation, mature adipocytes were washed with 150 µL of lipolysis wash buffer and incubated with 150 µL of lipolysis assay buffer containing COS at 320, 640, or 1280 µg/mL. For the stimulation of lipolysis, isoproterenol was added to a final concentration of 100 nM. After 12 h of incubation at 37 °C, 50 μL of the culture supernatant was transferred to a 96-well plate, and 50 μL of the reaction mixture (46 μL glycerol assay buffer, 2 μL enzyme mix, and 2 μL probe) was added. The plate was incubated for 30 min at room temperature in the dark, and glycerol release was quantified by measuring absorbance at 570 nm. Glycerol concentration was calculated using a standard curve and expressed as nmol glycerol/well.

### 4.7. Triglyceride Quantification

Intracellular triglyceride (TG) content was quantified using a Triglyceride Assay Kit (ab65336, Abcam, Cambridge, UK) according to the manufacturer’s instructions. Briefly, 3T3-L1 cells treated with or without COS in adipogenic induction medium for 12 days were washed with PBS, and cell pellets were resuspended and homogenized in 5% NP-40. To solubilize triglycerides, homogenates were slowly heated to 80–100 °C in a water bath for 2–5 min until the solution became cloudy, then cooled to room temperature; this heating–cooling cycle was repeated once to ensure complete solubilization. Insoluble material was removed by centrifugation, and 50 µL of the supernatant was loaded into each well of a 96-well plate. Cholesterol esterase (2 µL) was added to each well, mixed gently, and incubated at room temperature for 20 min with continuous agitation to enzymatically hydrolyze triglycerides into glycerol and free fatty acids. Reaction master mix (50 µL) was then added per well, mixed gently, and incubated at room temperature for 60 min in the dark. Absorbance was measured immediately at 570 nm using a microplate reader. Triglyceride concentrations were calculated from a standard curve and normalized to total protein content, expressed as nmol/mg protein.

### 4.8. Glucose Uptake Determination

Glucose uptake in 3T3-L1 adipocytes was measured using a Glucose Uptake Assay Kit (MAK542, Sigma-Aldrich, Merck KGaA, Darmstadt, Germany) according to the manufacturer’s instructions. Cells (2 × 10^3^ per well) were seeded in a 96-well plate and differentiated for 12 days as described above. The medium was removed and cells were starved in serum-free medium overnight. Cells were washed and 90 µL of glucose uptake buffer was added to each well for 1 h. Cells were then stimulated with insulin at a final concentration of 1 µM and COS at 320, 640, or 1280 µg/mL. Cells were incubated for 20 min at 37 °C. Glucose uptake was initiated by adding 10 µL of 2-DG solution to each well. After 40 min of incubation, the reaction solution was removed, and cells were washed before lysis by the addition of 25 µL of acidic lysis buffer. Subsequently, 25 µL of neutralization buffer was added to each well, mixed thoroughly, and left at room temperature for 5–10 min. A total of 50 µL of 2-DG uptake assay working solution was added to each well. The plate was incubated at room temperature for 2 h in the dark, and absorbance was measured using a microplate reader at 570–610 nm. Glucose uptake was expressed as % 2-DG6P accumulation relative to the insulin-stimulated control.

### 4.9. Protein Extraction and Western Blot Analysis

For protein extraction, 3T3-L1 cells (1 × 10^5^ cells/well) were seeded in 6-well culture plates. After reaching confluence for 3–4 days, cells were treated with adipogenic induction medium and COS at 320, 640, and 1280 µg/mL for 12 days. Following incubation, cells were rinsed with PBS and lysed in RIPA buffer (20 mM Tris-HCl pH 7.5, 150 mM NaCl, 1 mM Na_2_EDTA, 1 mM EGTA, 1% NP-40, 1% sodium deoxycholate, 2.5 mM sodium pyrophosphate, 1 mM β-glycerophosphate, 1 mM Na_3_VO_4_, 1 µg/mL leupeptin, 1 mM PMSF, and 20 mM NaF; Cell Signaling Technology, Danvers, MA, USA) for 5 min on ice, followed by brief sonication (3 × 5 s pulses on ice). The lysate was centrifuged and the supernatant was collected. Protein concentrations were determined using a Pierce BCA Protein Assay Kit (Thermo Fisher Scientific, Waltham, MA, USA).

Protein samples (20 µg per lane) were heated at 95 °C for 10 min, separated by 12.5% SDS-polyacrylamide gel electrophoresis (SDS-PAGE), and transferred onto a 0.45 µm nitrocellulose membrane (Cytiva, Marlborough, MA, USA). Membranes were blocked with 5% non-fat dry milk in 1x TBS-T (TBS containing 0.1% Tween-20) for 2 h at room temperature, then probed overnight at 4 °C with primary antibodies against PPARγ (1:1000; Cell Signaling Technology, Danvers, MA, USA), C/EBPα (1:1000; Cell Signaling Technology, Danvers, MA, USA), and β-actin (1:5000; Proteintech Group, Inc., Rosemont, IL, USA). Membranes were then incubated with dilution a 1:10000 of the anti-rabbit HRP-conjugated secondary antibody (Proteintech Group, Inc., Rosemont, IL, USA) for 1 h at room temperature. Protein bands were detected using an ECL chemiluminescence detection reagent (Bio-Rad Laboratories, Hercules, CA, USA) and imaged with an Amersham Imager 600 (Cytiva, Marlborough, MA, USA). Band intensities were quantified using ImageJ software (version 1.53, National Institutes of Health, Bethesda, MD, USA) and expressed as the ratio of target protein to β-actin.

### 4.10. Statistical Analysis

Statistical analysis was performed using GraphPad Prism Software (version 10.0, GraphPad Software, La Jolla, CA, USA). Data are presented as mean ± SEM. Differences among multiple groups were analyzed by one-way ANOVA followed by Tukey’s multiple comparisons test. Statistical significance is indicated as follows: asterisks (*) denote significant differences compared to the non-differentiated control group, and hash symbols (#) denote significant differences compared to the differentiated control group (without COS treatment), where (*) or (#) *p* < 0.05; (**) or (##) *p* < 0.01; (***) or (###) *p* < 0.001; and (****) or (####) *p* < 0.0001. All in vitro experiments were performed in at least four independent replicates.

### 4.11. Transcriptomic Analysis of Adipogenic Differentiation Gene Expression

To determine the effect of COS on gene expression, 3T3-L1 preadipocytes were cultured in an adipogenic induction medium supplemented with COS at 1280 µg/mL for 12 days. At the designated time, total RNA was isolated from three independent biological replicates per condition (Adipo diff and Adipo diff + COS 1280 µg/mL), each derived from separate 3T3-L1 cultures, using TriPure™ Isolation reagent (Roche, Mannheim, Germany). The concentration and purity of each sample were measured as described above. RNA samples from the three biological replicates within each condition were pooled in equal proportions before library preparation. The pooled RNA samples were submitted to BGI Genomics (BGI, New Territories, Hong Kong) for the evaluation of RNA integrity number (RIN) using an Agilent 2100 Bioanalyzer (Agilent, Santa Clara, CA, USA) and mRNA-seq utilizing the DNBseq sequencing platform, and the read length was 150 bp. The sequencing data were filtered using SOAPnuke software (version 2.3) by removing read sequence adapters, low-quality base ratios, and unknown bases. The clean filtered reads were aligned to the mouse reference genome using HISAT2 software (version 2.0.4). Bowtie2 (version 2.2.5) was applied to align the clean reads to the gene set. Fragments per kilobase per million map reads (FPKM) and TPM (transcript per million) values were calculated to evaluate the expression level by RSEM (version 1.2.8). All 3T3-L1 preadipocytes under control and treatment transcripts were submitted and available in the NCBI database with the accession number PRJNA1444491 at https://www.ncbi.nlm.nih.gov/sra/PRJNA1444491 (accessed on 28 March 2026).

The functional analyses of the DEGs under the adipogenic induction of 3T3-L1 preadipocytes were performed in the presence or absence of COS. Differentially expressed genes (DEGs) were identified using DESeq2. The significant DEGs were defined based on a Q value (adjusted *p* value) ≤ 0.05 and absolute log2-fold change (FC) ≥ 0.2. To gain insight into the change in phenotype, GO (http://www.geneontology.org/ (accessed on 7 March 2026)) and KEGG (https://www.kegg.jp/ (accessed on 7 March 2026)) enrichment analysis of annotated different expression genes was performed by Phyper based on the hypergeometric test. The significant levels of terms and pathways were corrected by Q value with a rigorous threshold (Q value ≤ 0.05). All the transcriptomic analysis was performed using the Dr. Tom program (https://biosys.bgi.com (accessed on 7 March 2026)) to investigate the significant biological functions and pathways.

## 5. Conclusions

This study provides comprehensive in vitro evidence that COS exerts anti-adipogenic effects in 3T3-L1 preadipocytes through a multi-target mechanism. Notably, a simple acid hydrolysis protocol produced a COS fraction of low molecular weight, DP2 at *m*/*z* 344.79 (below 1 kDa) is proposed to facilitate cellular uptake and intracellular bioactivity. Functionally, this COS fraction dose-dependently reduced lipid accumulation, triglyceride content, and adipocyte maturation while enhancing lipolysis and insulin-mediated glucose uptake at non-cytotoxic concentrations. At the molecular level, Western blot analysis confirmed the dose-dependent downregulation of the master adipogenic transcription factors PPARγ and C/EBPα at the protein level, consistent with the inhibition of downstream lipogenic targets previously reported in COS-treated adipocyte models. RNA-seq transcriptomic profiling further provided a gene expression dataset of COS-treated 3T3-L1 adipocytes, revealing coordinated transcriptional changes across the PPAR signaling, PI3K-Akt, AMPK, insulin signaling, adipocytokine signaling, and fatty acid metabolism pathways, offering a molecular basis for the observed anti-adipogenic effects. These findings provide transcriptomic insight into the potential molecular mechanisms underlying the observed anti-adipogenic effects of COS in vitro. While previous in vivo studies have demonstrated the beneficial metabolic effects of COS in high-fat diet models, the present study extends current knowledge by providing a detailed transcriptomic characterization of low-molecular-weight COS-mediated responses during adipocyte differentiation in 3T3-L1 cells. Future studies should investigate the bioavailability and in vivo efficacy of well-defined low-molecular-weight COS fractions in obesity animal models to validate the translational relevance of these findings.

## Figures and Tables

**Table 1:** Characterization of COS components by using MALDI-TOF MS. ‘M’ denotes the composition of COS, while bold letters indicate the major compounds.

*m*/*z*	Ion Type	DP	Ion Composition
Observed			
**344.7934**	**[M + H]** ^+^	2	(GlcN)_2_
**361.7861**	**[M + NH_4_]** ^+^	2	(GlcN)_2_
383.7568	[M + H]^+^	2	(GlcN)(GlcNAc)
408.8480	[M + Na]^+^	2	(GlcN)(GlcNAc)
429.0150	[M + Na]^+^	2	(GlcNAc)_2_ dehydrated
**441.0675**	**[M + NH_4_]** ^+^	2	(GlcNAc)_2_
457.0305	[M + Na]^+^	2	(GlcNAc)_2_
469.0675	[M + Na]^+^	2	(GlcNAc)_2_ hydrated
**518.7361**	**[M + NH4]** ^+^	3	(GlcN)_3_
578.7786	[M + Na]^+^	3	(GlcN)_2_(GlcNAc) hydrated
**626.8980**	**[M + H]** ^+^	3	(GlcNAc)_3_
660.6845	[M + H]^+^	4	(GlcN)_4_
768.8350	[M + Na]^+^	4	(GlcN)_2_(GlcNAc)_2_
810.8140	[M + Na]^+^	4	(GlcN)(GlcNAc)_3_
929.7855	[M + Na]^+^	5	(GlcN)_3_(GlcNAc)_2_
971.7540	[M + Na]^+^	5	(GlcN)_2_(GlcNAc)_3_
1013.7294	[M + Na]^+^	5	(GlcN)(GlcNAc)_4_
1055.7060	[M + Na]^+^	5	(GlcNAc)_5_
1090.7177	[M + Na]^+^	6	(GlcN)_4_(GlcNAc)_2_
1132.6915	[M + Na]^+^	6	(GlcN)_3_(GlcNAc)_3_
1174.6603	[M + Na]^+^	6	(GlcN)_2_(GlcNAc)_4_
1216.6334	[M + Na]^+^	6	(GlcN)(GlcNAc)_5_
1258.6098	[M + Na]^+^	6	(GlcNAc)_6_
1335.5821	[M + Na]^+^	7	(GlcN)_3_(GlcNAc)_4_
1377.5616	[M + Na]^+^	7	(GlcN)_2_(GlcNAc)_5_
1420.5184	[M + Na]^+^	7	(GlcN)(GlcNAc)_6_
1461.5138	[M + Na]^+^	7	(GlcNAc)_7_
1496.5080	[M + Na]^+^	8	(GlcN)_4_(GlcNAc)_4_
1538.4648	[M + Na]^+^	8	(GlcN)_3_(GlcNAc)_5_
1580.4290	[M + Na]^+^	8	(GlcN)_2_(GlcNAc)_6_
1664.3539	[M + Na]^+^	8	(GlcNAc)_8_
1742.3592	[M + Na]^+^	9	(GlcN)_3_(GlcNAc)_6_
1783.3044	[M + Na]^+^	9	(GlcN)_2_(GlcNAc)_7_
1825.2544	[M + Na]^+^	9	(GlcN)GlcNAc)_8_
1869.2652	[M + Na]^+^	9	(GlcNAc)_9_

**Table 2:** The differential expression of genes related to PPAR signaling pathway/PI3K-Akt signaling pathway and insulin signaling pathway.

Expression	Pathway	Gene Name	Gene Description	Accession Number	Log_2_FC (AdipoCOS/Adipo)	*p*-Value (AdipoCOS/Adipo)	FDR (AdipoCOS/Adipo)
Upregulation	PPAR signaling pathway	*Acox2*	Acyl-coA oxidase 2	NM_053115	10.10	4.78 × 10^−4^	2.23 × 10^−3^
	PPAR signaling pathway	*Rxrb*	Retinoid X receptor beta	NM_001205214	2.51	1.32 × 10^−16^	3.36 × 10^−15^
	PPAR signaling pathway	*Nr1h3*	Nuclear receptor subfamily 1 group H member 3	NM_001177730	2.23	1.36 × 10^−5^	8.94 × 10^−5^
	PPAR signaling pathway/PI3K-Akt signaling pathway	*Pdpk1*	3-Phosphoinositide Dependent Protein Kinase 1	NM_001286662	1.73	3.13 × 10^−62^	6.26 × 10^−60^
	PPAR signaling pathway	*Gk*	Glycerol kinase	NM_001294140	1.46	1.05 × 10^−5^	7.01 × 10^−5^
	Fatty acid degradation/AMPK signaling pathway	*Cpt1c*	Carnitine palmitoyltransferase 1C	NM_001357670	1.45	8.95 × 10^−4^	3.89 × 10^−3^
	Fatty acid metabolism	*Hmgcs2*	3-hydroxy-3-methylglutaryl-CoA synthase 1	NM_008256	0.93	1.60 × 10^−4^	8.36 × 10^−4^
	PPAR signaling pathway	*Acox3*	Acyl-coA oxidase 3	NM_030721	0.56	4.74 × 10^−5^	2.78 × 10^−4^
	PPAR signaling pathway	*Plin4*	Perilipin 4	NM_030721	0.37	2.35 × 10^−8^	2.47 × 10^−7^
Downregulation	PPAR signaling pathway/Insulin signaling pathway	*Sorbs1*	Sorbin and SH3 domain containing 1	NM_001034963	−11.71	1.24 × 10^−10^	1.73 × 10^−9^
	PPAR signaling pathway	*Aqp7*	Aquaporin 7	NM_007473	−3.17	1.19 × 10^−2^	3.68 × 10^−2^
	PPAR signaling pathway	*Acsl3*	Acyl-CoA synthetase long chain family member 3	NM_028817	−2.23	1.18 × 10^−36^	1.00 × 10^−34^
	PPAR signaling pathway	*Slc27a1*	Solute carrier family 27 member 1	NM_001357180	−1.41	2.03 × 10^−8^	2.14 × 10^−7^
	PPAR signaling pathway	*Ilk*	Integrin linked kinase	NM_010562	−0.71	8.30 × 10^−15^	1.81 × 10^−13^
	PPAR signaling pathway	*Plin1*	Perilipin 1	NM_001113471	−0.69	1.66 × 10^−2^	4.80 × 10^−2^
	PPAR signaling pathway	*Rxrb*	Retinoid X receptor beta	NM_011306	−0.54	4.57 × 10^−3^	1.61 × 10^−2^
	PPAR signaling pathway/AMPK signaling pathway	*Pparg*	Peroxisome proliferator-activated receptor gamma	NM_001308354	−0.46	6.45 × 10^−4^	2.90 × 10^−3^
	PPAR signaling pathway	*Fabp5*	Fatty acid-binding protein 5	NM_010634	−0.39	1.13 × 10^−29^	7.07 × 10^−28^
	PPAR signaling pathway	*Fads2*	Phosphoenolpyruvate carboxykinase 2	NM_019699	−0.35	5.58 × 10^−17^	1.48 × 10^−15^

**Table 3:** The differential expression of genes related to the AMPK pathway/insulin signaling pathway and PI3K/Akt signaling pathway.

Expression	Pathway	Gene Name	Gene Description	Accession Number	Log_2_FC (AdipoCOS/Adipo)	*p*-Value (AdipoCOS/Adipo)	FDR (AdipoCOS/Adipo)
Upregulation	AMPK signaling pathway/Insulin signaling pathway/PI3K-Akt signaling pathway	*Akt2*	AKT serine/threonine kinase 2	NM_001331109	14.28	1.73 × 10^−60^	3.28 × 10^−58^
	AMPK signaling pathway/Insulin signaling pathway/PI3K-Akt signaling pathway	*Tsc1*	Tuberous Sclerosis Complex 1	NM_001289575	12.73	5.99 × 10^−21^	2.10 × 10^−19^
	AMPK signaling pathway/Insulin signaling pathway/PI3K-Akt signaling pathway	*Prkaa2*	Protein kinase, AMP-activated, catalytic subunit alpha 2	NM_001356568	11.81	1.36 × 10^−11^	2.12 × 10^−10^
	AMPK signaling pathway/PI3K-Akt signaling pathway	*Creb3l4*	Cyclic AMP-responsive element-binding protein 3-like protein 4	NM_001307934	9.45	7.70 × 10^−3^	2.53 × 10^−2^
	AMPK signaling pathway	*Sirt1*	Sirtuin 1	NM_001159589	9.42	1.54 × 10^−2^	4.56 × 10^−2^
	AMPK signaling pathway/Insulin signaling pathway	*Prkag2*	Protein Kinase AMP-Activated Non-Catalytic Subunit Gamma 2	NM_145401	1.98	1.07 × 10^−4^	5.82 × 10^−4^
	AMPK signaling pathway	*Cab39*	Calcium-Binding Protein 39	NM_001355047	1.17	1.65 × 10^−14^	3.47 × 10^−13^
	AMPK signaling pathway	*Ulk1*	Unc-51 like autophagy activating kinase 1	NM_001347394	1.03	3.41 × 10^−8^	3.47 × 10^−7^
	AMPK signaling pathway/PI3K-Akt signaling pathway	*Ppp2r3a*	Protein Phosphatase 2A Regulatory Subunit B56δ	NM_172144	086	2.76 × 10^−3^	1.05 × 10^−2^
Downregulation	AMPK signaling pathway/PI3K-Akt signaling pathway	*Igf1*	Insulin-like Growth Factor 1	NM_001111276	−11.93	1.96 × 10^−12^	3.36 × 10^−11^
	AMPK signaling pathway/PI3K-Akt signaling pathway	*Ppp2r5c*	Protein Phosphatase 2 Regulatory Subunit B″Gamma	NM_001135001	−9.70	3.55 × 10^−63^	7.34 × 10^−61^
	AMPK signaling pathway/Insulin signaling pathway/PI3K-Akt signaling pathway	*Pik3cd*	Phosphatidylinositol-4,5-bisphosphate 3-kinase catalytic subunit delta	NM_008840	−2.63	1.08 × 10^−4^	5.84 × 10^−4^
	AMPK signaling pathway/Insulin signaling pathway	*Srebf1*	Sterol Regulatory Element-Binding Transcription Factor 1	NM_001313979	−2.20	1.52 × 10^−8^	1.64 × 10^−7^
	AMPK signaling pathway/Insulin signaling pathway/PI3K-Akt signaling pathway	*Pik3r3*	Phosphatidylinositol 3-kinase regulatory subunit gamma	NM_001355584	−0.59	7.98 × 10^−3^	2.59 × 10^−2^
	AMPK signaling pathway	*Akt1s1*	Proline-rich AKT1 substrate 1	NM_001290694	−0.54	2.22 × 10^−3^	8.63 × 10^−3^
	AMPK signaling pathway/PI3K-Akt signaling pathway	*Pik3r1*	Phosphatidylinositol 3-kinase, regulatory subunit, polypeptide 1	NM_001024955	−0.45	2.22 × 10^−6^	1.68 × 10^−5^
	AMPK signaling pathway/Insulin signaling pathway	*Lipe*	Lipase E	NM_010719	−0.41	2.10 × 10^−3^	8.22 × 10^−3^

**Table 4:** Differential upregulation of gene expression related to PI3K/Akt signaling pathway.

Expression	Pathway	Gene Name	Gene Description	Accession Number	Log_2_FC (AdipoCOS/Adipo)	*p*-Value (AdipoCOS/Adipo)	FDR (AdipoCOS/Adipo)
Upregulation	PI3K-Akt signaling pathway	*Bcl2l11*	Bcl-2-like protein 11	NM_009754	11.91	3.39 × 10^−12^	5.68 × 10^−11^
	PI3K-Akt signaling pathway	*Lpar1*	Lysophosphatidic acid receptor 1,	NM_001290486	11.88	6.79 × 10^−12^	1.10 × 10^−10^
Downregulation	PI3K-Akt signaling pathway	*Pkn1*	Protein kinase N1	NM_001199593	−14.08	2.29 × 10^−52^	3.40 × 10^−50^
	PI3K-Akt signaling pathway	*Ywhaz*	Tyrosine 3-monooxygenase/tryptophan 5-monooxygenase activation protein zeta	NM_001253806	−12.50	7.71 × 10^−18^	2.17 × 10^−16^
	PI3K-Akt signaling pathway	*Fgfr3*	Fibroblast growth factor receptor 3	NM_008010	−11.29	6.24 × 10^−8^	6.14 × 10^−7^
	PI3K-Akt signaling pathway	*Ptk2*	Protein tyrosine kinase 2	NM_001358046	−11.04	4.96 × 10^−7^	4.24 × 10^−6^
	PI3K-Akt signaling pathway	*Egf*	Epidermal growth factor	NM_001329594	−10.93	1.98 × 10^−6^	1.52 × 10^−5^
	PI3K-Akt signaling pathway	*Bdnf*	Brain-derived neurotrophic factor	NM_007540	−9.23	1.58 × 10^−2^	4.63 × 10^−2^
	PI3K-Akt signaling pathway	*Vegfa*	Vascular endothelial growth factor A	NM_001110268	−0.89	2.26 × 10^−5^	1.42 × 10^−4^

**Table 5:** Differential upregulation of gene expression related to insulin signaling pathway.

Expression	Pathway	Gene Name	Gene Description	Accession Number	Log_2_FC (AdipoCOS/Adipo)	*p*-Value (AdipoCOS/Adipo)	FDR (AdipoCOS/Adipo)
Upregulation	Insulin signaling pathway	*Mapk8*	Mitogen-activated protein kinase 8	NM_001310452	0.56	2.69 × 10^−5^	3.47 × 10^−7^
	Insulin signaling pathway	*Mapk1*	Mitogen-activated protein kinase 1	NM_001357115	0.30	1.18 × 10^−6^	9.39 × 10^−6^
Downregulation	Insulin signaling pathway	*Phka1*	Phosphorylase kinase regulatory subunit alpha 1	NM_173021	−11.89	7.80 × 10^−12^	1.25 × 10^−10^
	Insulin signaling pathway	*Socs2*	Suppressor of cytokine signaling 2	NM_001168656	−9.64	7.92 × 10^−3^	2.58 × 10^−2^
	Insulin signaling pathway	*Mapk9*	Mitogen-activated protein kinase 9	NM_001163671	−5.07	8.83 × 10^−20^	2.90 × 10^−18^
	Insulin signaling pathway	*Exoc7*	Exocyst complex component 7	NM_001347636	−3.42	2.17 × 10^−5^	1.36 × 10^−4^
	Insulin signaling pathway	*Map2k2*	Mitogen-activated protein kinase kinase 2	NM_001358539	−1.44	2.15 × 10^−4^	1.10 × 10^−3^
	Insulin signaling pathway	*Hk1*	Hexokinase 1	NM_010438	−0.87	4.10 × 10^−3^	1.46 × 10^−2^

## Data Availability

The original contributions presented in this study are included in this article. Further inquiries can be directed to the corresponding author.

## Supplementary Materials

The following supporting information can be downloaded at: https://www.mdpi.com/article/10.3390/ijms27114970/s1.

## Abbreviations

The following abbreviations are used in this manuscript:

COS	Chitosan Oligosaccharides
MALDI-TOF	Matrix-Assisted Laser Desorption/Ionization Time-of-Flight
NMR	Nuclear Magnetic Resonance
DP	Degree of polymerization
DD	Degrees of deacetylation
GO	Gene Ontology
KEGG	Kyoto Encyclopedia of Genes and Genomes
DEGs	Differentially expressed genes
ATCC	American Type Culture Collection
