# Supplementary material for: Chitosan Oligosaccharides Suppress Adipogenesis and Lipid Accumulation in 3T3-L1 Preadipocytes via Multi-Pathway Transcriptomic Reprogramming
Source: Int J Mol Sci. 2026 May 30;27(11):4970. doi: 10.3390/ijms27114970 (PMC13256913; doi:10.3390/ijms27114970)
Supplement: Supplementary file 1 [file ijms-27-04970-s001.zip › Supplementary file 1 for submission.pdf]

International Journal of *Molecular Sciences*

MDPI

Article

Chitosan Oligosaccharides Suppress Adipogenesis and Lipid Accumulation in 3T3-L1 Preadipocytes via Multi-Pathway Transcriptomic Reprogramming

Sineenart Songkoomkrong [1,2], Siriporn Nonkhwao [1,2], Jirawat Saetan [3], Supawadee Duangprom [1,2], Prateep Amonruttanapun [1,2], Piyapon Janpan [1,2], Prasert Sobhon [4] and Napamanee Kornthong [1,2,*]

1 Chulabhorn International College of Medicine, Thammasat University, Rangsit Campus, Pathumthani 12120, Thailand
2 Research Unit in Innovative Marine Biotechnology and Natural Bio-Resources for Sustainable Health and Wellness, Thammasat University, Pathumthani 12120, Thailand
3 Division of Health and Applied Sciences, Faculty of Science, Prince of Songkla University, Hat Yai, Songkhla 90110, Thailand
4 Department of Anatomy, Faculty of Science, Mahidol University, Bangkok 10400, Thailand
* Correspondence: napamaneenatt@gmail.com or napanatt@tu.ac.th

Academic Editor: Rosemary Kiernan

Received: 7 April 2026
Revised: 27 May 2026
Accepted: 28 May 2026
Published: 30 May 2026

Copyright: © 2026 by the authors. Submitted for possible open access publication under the terms and conditions of the Creative Commons Attribution (CC BY) license.

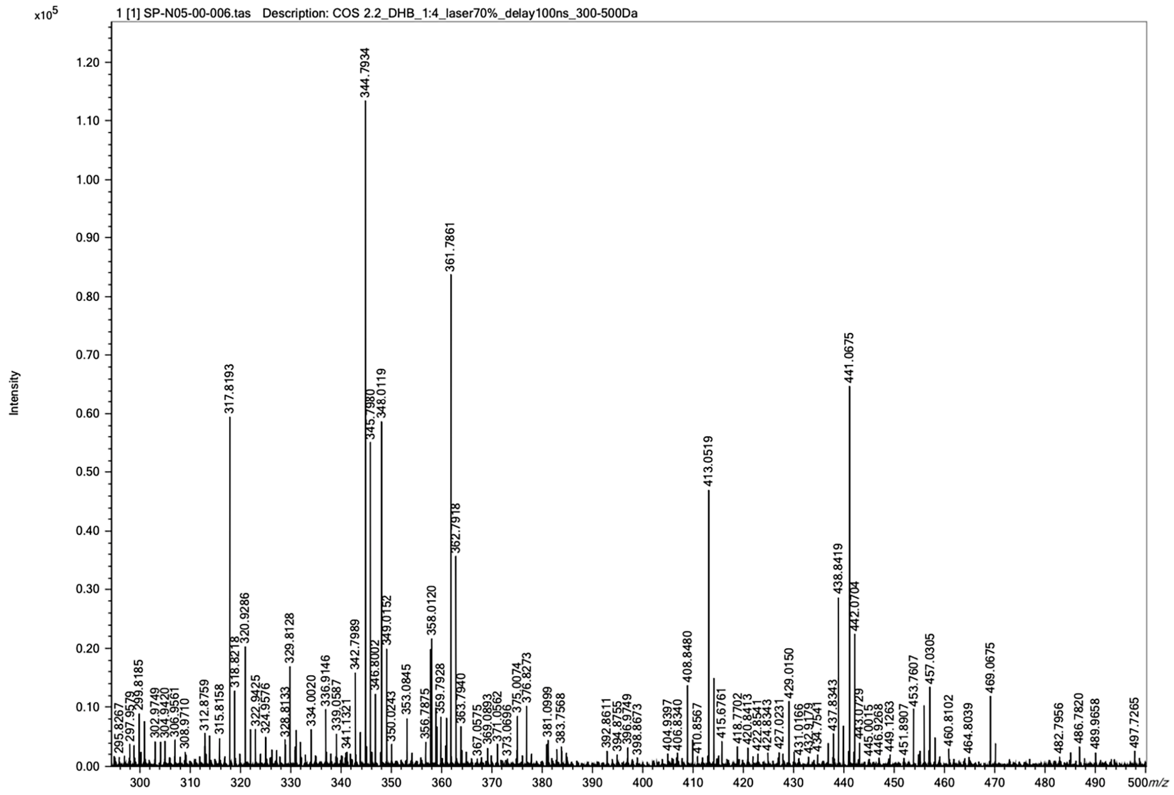

Figure S1. Positive ion MALDI-TOF MS mass spectrum of the mixture of COS (with DHB matrix) in the range 300 to 500 Da.

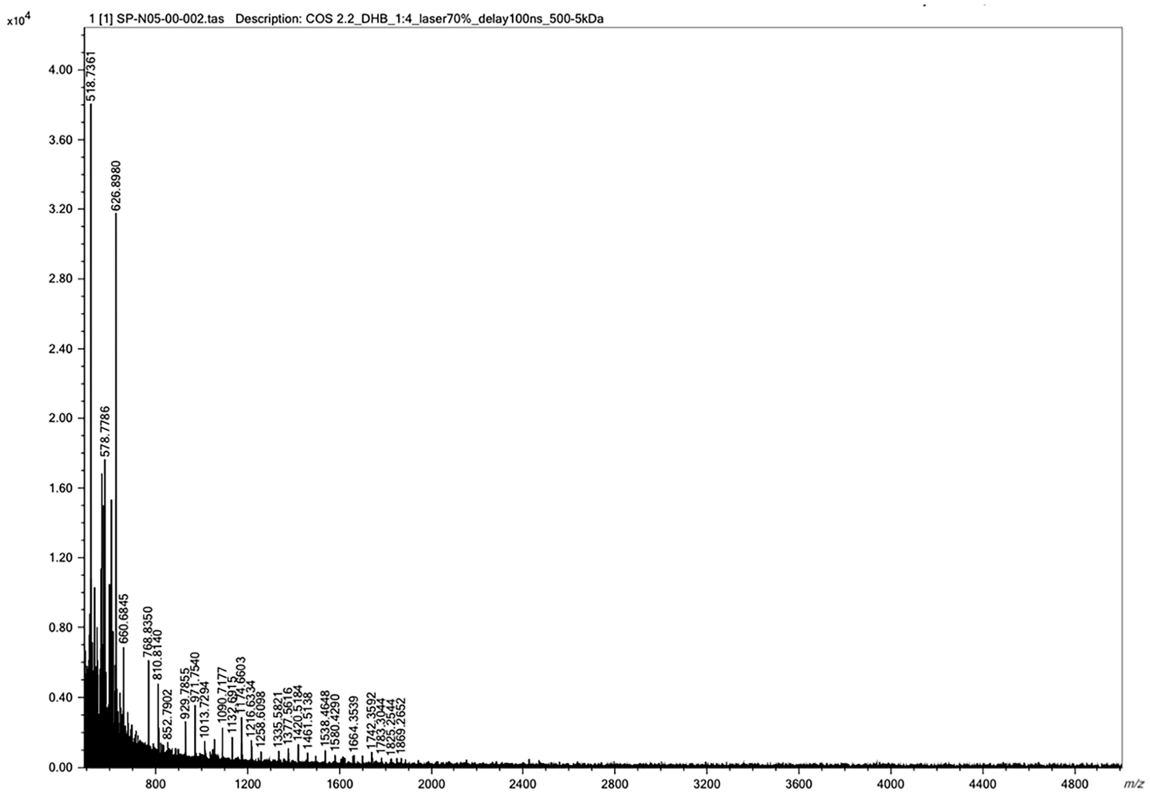

Figure S2. Positive ion MALDI-TOF MS mass spectrum of the mixture of COS (with DHB matrix) in the range 500 to 5000 Da.

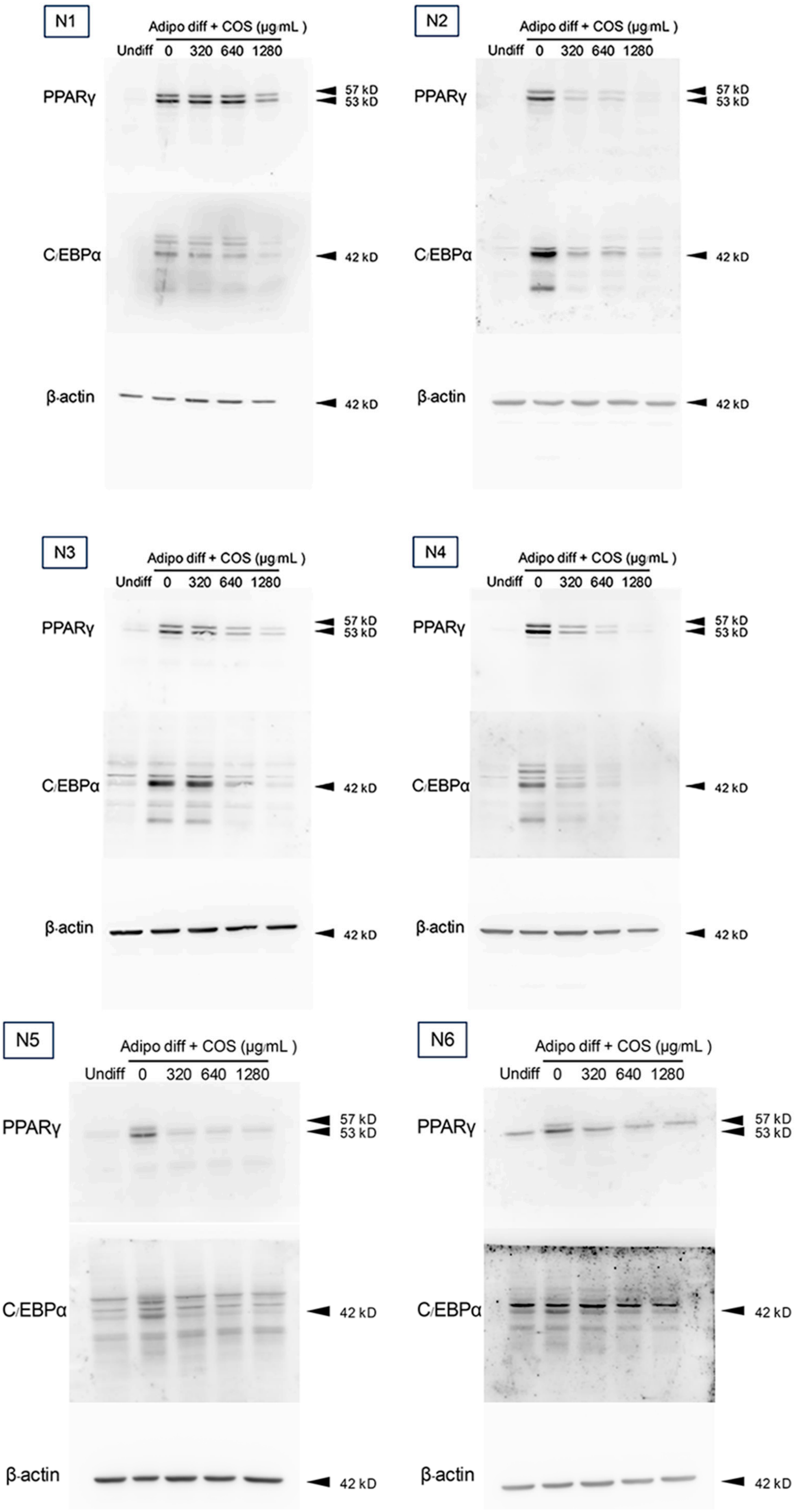
N1
Adipo diff + COS (μg/mL)
Undiff 0 320 640 1280
PPARγ
57 kD
53 kD
C/EBPα
42 kD
β-actin
42 kD
N2
Adipo diff + COS (μg/mL)
Undiff 0 320 640 1280
PPARγ
57 kD
53 kD
C/EBPα
42 kD
β-actin
42 kD
N3
Adipo diff + COS (μg/mL)
Undiff 0 320 640 1280
PPARγ
57 kD
53 kD
C/EBPα
42 kD
β-actin
42 kD
N4
Adipo diff + COS (μg/mL)
Undiff 0 320 640 1280
PPARγ
57 kD
53 kD
C/EBPα
42 kD
β-actin
42 kD
N5
Adipo diff + COS (μg/mL)
Undiff 0 320 640 1280
PPARγ
57 kD
53 kD
C/EBPα
42 kD
β-actin
42 kD
N6
Adipo diff + COS (μg/mL)
Undiff 0 320 640 1280
PPARγ
57 kD
53 kD
C/EBPα
42 kD
β-actin
42 kD

Figure S3. The western blot results of the PPARγ, C/EBPα and β-actin protein expression in 3T3-L1 after cells were treated with and without COS in adipogenic induction medium for 12 days. The level of protein expression was analyzed from 6 independent experiments.

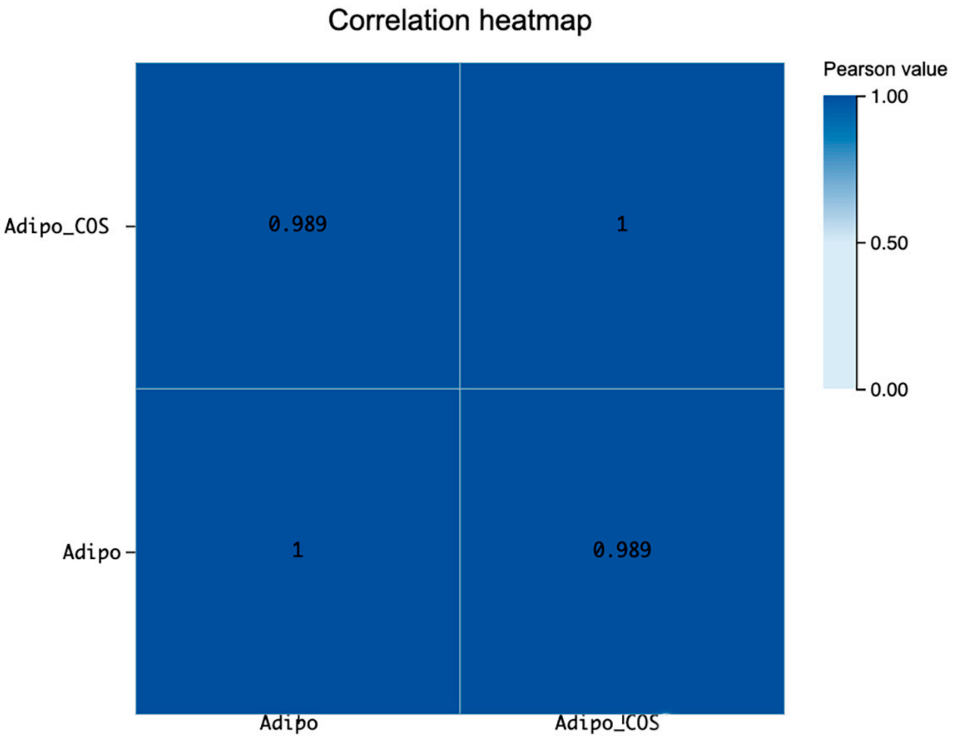

Figure S4. The Pearson correlation coefficients of all gene expression between biological replicates. These coefficients were reflected in the form of a heat map.

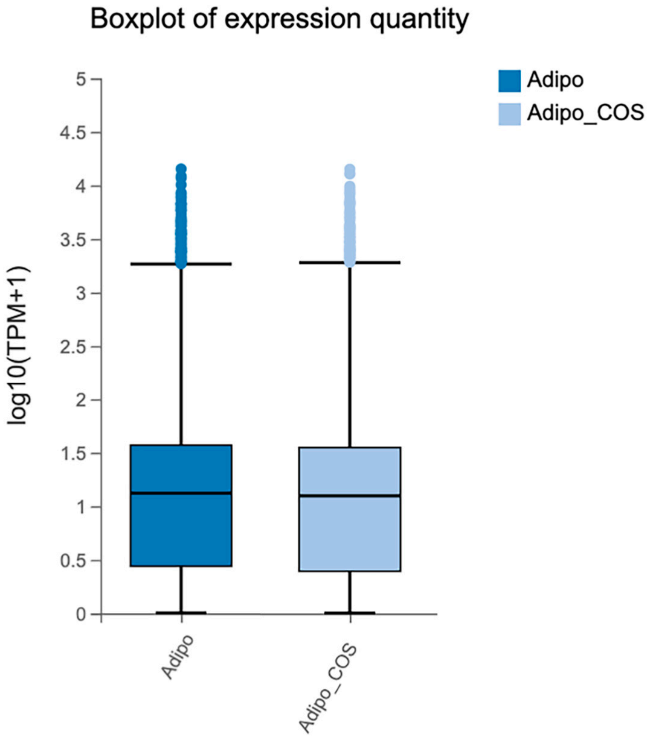

Figure S5. The boxplots of gene expression levels in each sample.

https://doi.org/10.3390/ijms27114970

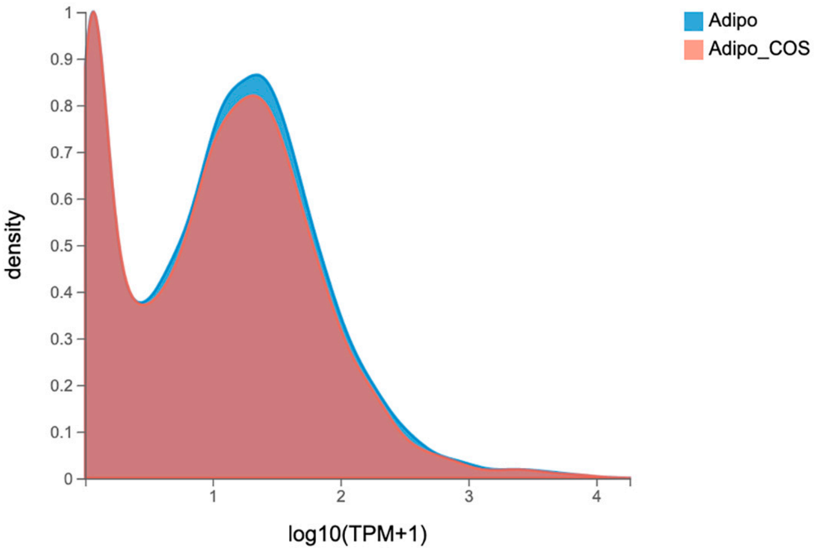

Figure S6. The density map of gene abundance in each sample.

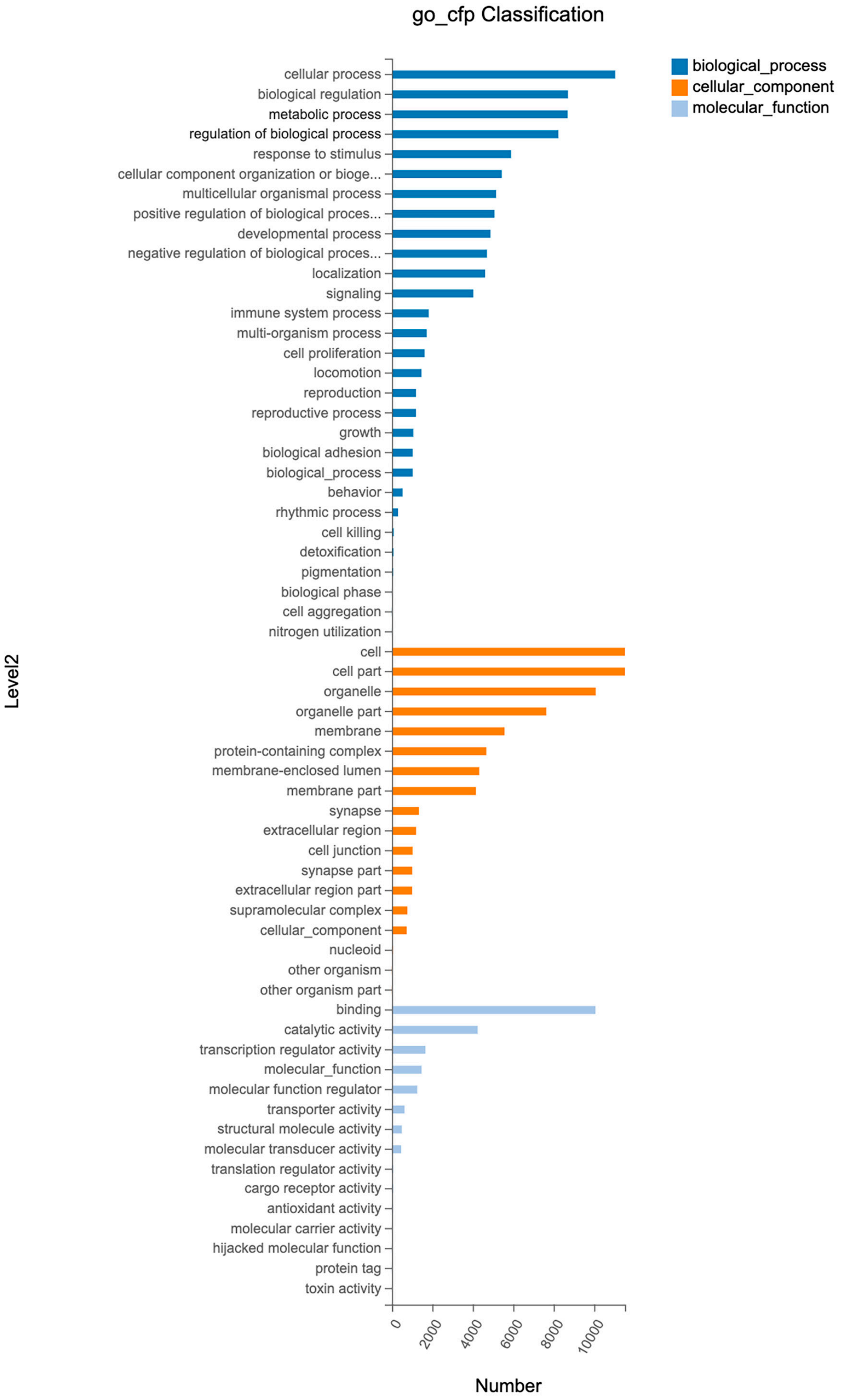

Figure S7. The top enriched GO terms across cellular component (CC), molecular function (MF), and biological process (BP) categories highlighted processes central to adipocyte biology.

Disclaimer/Publisher's Note: The statements, opinions and data contained in all publications are solely those of the individual author(s) and contributor(s) and not of MDPI and/or the editor(s). MDPI and/or the editor(s) disclaim responsibility for any injury to people or property resulting from any ideas, methods, instructions or products referred to in the content.

https://doi.org/10.3390/ijms27114970
